# Performance Analysis of Explainable Deep Learning-Based Intrusion Detection Systems for IoT Networks: A Systematic Review

**DOI:** 10.3390/s26020363

**Published:** 2026-01-06

**Authors:** Taiwo Blessing Ogunseyi, Gogulakrishan Thiyagarajan, Honggang He, Vinay Bist, Zhengcong Du

**Affiliations:** 1 School of Electronic and Information Engineering, Yibin University, Yibin 644000, China; 2 Software Engineering, Cisco Systems Inc., Austin, TX 78759, USA; gogs.ethics@gmail.com; 3 School of Mechanical and Electrical Engineering, Yibin University, Yibin 644000, China; hehonggang@yibinu.edu.cn; 4 Engineering Department, Dell Technologies, Leander, TX 78641, USA; vinay.bist@gmail.com

**Keywords:** performance analysis, explainable deep learning, intrusion detection systems, internet of things, evaluation metrics

## Abstract

The opaque nature of black-box deep learning (DL) models poses significant challenges for intrusion detection systems (IDSs) in Internet of Things (IoT) networks, where transparency, trust, and operational reliability are critical. Although explainable artificial intelligence (XAI) has been increasingly adopted to enhance interpretability, its impact on detection performance and computational efficiency in resource-constrained IoT environments remains insufficiently understood. This systematic review investigates the performance of an explainable deep learning-based IDS for IoT networks by analyzing trade-offs among detection accuracy, computational overhead, and explanation quality. Following the PRISMA methodology, 129 peer-reviewed studies published between 2018 and 2025 are systematically analyzed to address key research questions related to XAI technique trade-offs, deep learning architecture performance, post-deployment XAI evaluation practices, and deployment bottlenecks. The findings reveal a pronounced imbalance in existing approaches, where high detection accuracy is often achieved at the expense of computational efficiency and rigorous explainability evaluation, limiting practical deployment on IoT edge devices. To address these gaps, this review proposes two conceptual contributions: (i) an XAI evaluation framework that standardizes post-deployment evaluation categories for explainability, and (ii) the Unified Explainable IDS Evaluation Framework (UXIEF), which models the fundamental trilemma between detection performance, resource efficiency, and explanation quality in IoT IDSs. By systematically highlighting performance–efficiency gaps, methodological shortcomings, and practical deployment challenges, this review provides a structured foundation and actionable insights for the development of trustworthy, efficient, and deployable explainable IDS solutions in IoT ecosystems.

## 1. Introduction

The Internet of Things (IoT) represents a rapidly evolving ecosystem of interconnected devices, including smart appliances, wearables, vehicles, and cyber–physical systems equipped with sensors, actuators, and network connectivity, enabling large-scale data exchange and autonomous operation. Recent studies indicate that IoT adoption continues to accelerate across domains such as smart homes, industrial automation, healthcare, and smart cities, leading to unprecedented growth in device density and data volume [1,2]. However, this expansion has simultaneously intensified security risks, as IoT systems expose a broad attack surface encompassing software-, network-, and device-level vulnerabilities [3,4]. Contemporary threat analyses show that adversaries increasingly exploit these weaknesses through sophisticated attack vectors, including distributed denial-of-service, botnets, and protocol-specific exploits targeting resource-constrained IoT devices [5,6]. Although numerous intrusion detection and mitigation techniques have been developed for conventional networks, many remain unsuitable for IoT environments due to heterogeneous protocols, limited computational resources, and highly dynamic traffic characteristics [7,8].

Intrusion detection systems (IDSs) are detection systems strategically positioned within a network or installed on a host to analyze traffic, detect any malicious activity, and report those malicious activities to allow the security operation center to keep a constant watch on current threats [9,10]. IDSs are particularly crucial for securing the IoT due to the unique challenges posed by the vast array of connected devices and the diverse nature of their communications [11]. As IoT devices proliferate, the attack surface for potential cyber threats expands significantly. These devices often operate with limited security measures, making them susceptible to various attacks, such as unauthorized access, data exfiltration, and denial of service [12,13]. A robust IDS can intelligently monitor network traffic from these devices, identifying anomalous behaviors that indicate potential intrusions or attacks. The importance of IDSs in IoT security is underscored by their ability to provide real-time visibility into network activity, enabling organizations to quickly respond to security incidents [11,14]. By detecting intrusions and alerting security teams immediately, IDSs help to mitigate the potential damage that could result from a successful attack. This approach could enhance the security posture of IoT ecosystems and help protect sensitive information.

However, traditional IDSs consume a lot of computational resources, such as space and memory, with a significant amount of computing power; hence, these traditional IDSs may not be efficient for IoT systems due to IoT constraints such as limited computing power, memory, and storage space [11,14,15]. Moreover, IoT networks generate an enormous amount of critical and complex traffic data that can be analyzed for threat detection. As the volume and complexity of IoT traffic continue to grow, traditional IDSs also struggle to maintain the necessary efficiency and accuracy in identifying sophisticated threats [16]. This necessitates the integration of deep learning (DL) techniques into IDSs, which offers promising solutions to overcome the limitations associated with conventional intrusion systems.

Deep learning, a powerful subset of machine learning, leverages multi-layer neural network architectures to model complex, non-linear relationships in large-scale data, enabling improved learning accuracy, adaptability, and robustness. Recent studies demonstrate that deep learning techniques have become central to addressing emerging challenges in IoT environments, including high-dimensional traffic analysis, resource optimization, and dynamic decision-making under constrained conditions [17,18]. In particular, advanced deep reinforcement learning models, such as distributed Deep Deterministic Policy Gradient (DDPG)-based approaches, have been successfully applied to IoT systems for efficient resource allocation and age-of-information minimization, highlighting the growing role of deep learning beyond traditional classification tasks [19]. This evolution is especially relevant in IoT networks, where heterogeneous devices generate diverse and temporally correlated data streams that demand scalable and intelligent learning frameworks. Deep learning models have shown strong capability in extracting meaningful representations from such complex data, making them well suited for tasks such as network monitoring, anomaly detection, and intelligent security analytics in modern IoT infrastructures [20].

The deployment of deep learning in IDSs represents a transformative approach to cybersecurity within IoT networks [21]. By leveraging deep learning algorithms, these advanced intrusion detection systems can efficiently process and analyze the complexities of IoT traffic, accommodating the multitude of devices and the varied behaviors they exhibit. The ability of deep learning models to adapt and learn from new patterns enables them to identify emerging threats in real time, thus enhancing the overall security posture of IoT networks [22]. As concerns about the vulnerability of IoT devices grow, the deployment of DL-enhanced IDSs emerges as a crucial strategy for safeguarding against increasingly sophisticated cyber threats [23].

While DL models can significantly enhance threat detection capabilities by quickly analyzing vast datasets and identifying anomalies, their effectiveness hinges not only on accuracy but also on the transparency of their decision-making processes [24,25]. In IoT environments, where the interconnectivity of devices often leads to complex attack surfaces, stakeholders require insights into how and why certain actions are taken by these systems. Without such insights, trust in automated detection mechanisms may be eroded, potentially resulting in hesitance to rely on these technologies in real-time security scenarios [8].

In critical infrastructures, where the consequences of undetected intrusions can be catastrophic, the ability to comprehend the underlying rationale of IDS alerts is paramount [26]. By bridging the gap between the opaque nature of conventional deep learning models and human understanding, explainable artificial intelligence (XAI) can enhance trust in DL-based IDSs in IoT security. The integration of explainability not only strengthens trust among users but also fosters adaptation, thus creating resilient defense mechanisms against evolving cyber threats [27]. The adoption of XAI in DL-based IDSs is essential for ensuring robust, transparent, and efficient cybersecurity strategies in the intricate and dynamic landscape of IoT environments.

### 1.1. Motivation

Deep learning (DL) models have demonstrated strong effectiveness in intrusion detection by automatically learning complex patterns from large-scale network traffic data and achieving high detection accuracy [28]. However, many of these models operate as black boxes, offering little to no insight into their internal decision-making processes. This lack of interpretability raises significant concerns in security-critical applications, where understanding why a model flags traffic as malicious is essential for trust, validation, and operational response [29].

In intrusion detection systems (IDSs), the opacity of black-box DL models can lead to serious practical challenges. Security analysts may struggle to validate alerts, investigate false positives, or understand missed detections, ultimately increasing response time and operational burden [30,31]. Furthermore, the inability to interpret model behavior complicates the identification of biases, dataset artifacts, and architectural weaknesses, limiting the adaptability of IDSs to evolving and sophisticated cyber threats [28]. As IoT environments grow in scale and complexity, these limitations become increasingly pronounced due to constrained resources, heterogeneous devices, and dynamic traffic patterns.

Explainable artificial intelligence (XAI) has emerged as a promising solution to address these challenges by providing interpretable insights into DL-based IDS decisions. By revealing feature importance, decision rationale, or model behavior, XAI enables security professionals to better understand detected threats, improve incident response, and build confidence in automated detection systems [32]. Despite growing interest in explainable DL for IDS and IoT security, existing surveys primarily focus on architectural designs or explainability techniques, with limited attention to performance trade-offs. In particular, the impact of explainability on detection accuracy, computational efficiency, and practical deployability in resource-constrained IoT networks remains insufficiently explored. This study addresses this gap through a performance-oriented review of explainable DL-based IDSs in IoT environments, highlighting how our review differs from prior surveys on DL-based IDS and XAI in cybersecurity.

Specifically, this study aims to fill this gap by providing a systematic analysis of the performance of explainable DL-based IDSs in IoT networks by evaluating XAI performance within the IoT networks. A performance-focused evaluation is needed to transition IoT security solutions from experimental to real-world deployment by prioritizing metrics like latency and energy use, ensuring they are computationally feasible for resource-constrained edge environments and aligning with efficient edge AI.

### 1.2. Contributions

In this paper, we present a comprehensive and systematic analysis of the existing studies that evaluate the performance of explainable DL models for network intrusion detection systems within IoT networks. Specifically, the contributions of this study are as follows:

(i) To comprehensively analyze the performance trade-offs associated with integrating different XAI techniques into deep learning-based IDSs for IoT networks by evaluating the impact of these techniques on key performance indicators.
(ii) To identify and compare the performance characteristics of various explainable deep learning architectures applied to IDSs in IoT by evaluating the detection accuracy and resource efficiency of these models.
(iii) To examine the effectiveness and reliability of XAI techniques after deployment by reporting on the evaluation methods utilized for XAI techniques within the IoT security context.
(iv) To develop two conceptual frameworks: the XAI evaluation framework and the Unified Explainable IDS Evaluation Framework (UXIEF). The former is aimed at standardizing evaluation categories for XAI while the latter visually models the fundamental tensions between detection performance, resource efficiency, and explanation quality in IoT IDSs.
(v) To identify critical limitations and challenges hindering the widespread adoption of explainable deep learning-based IDSs in IoT deployment by synthesizing evidence from the literature on such factors.

The organization of the paper is as follows. Section 2 introduces related works on IDSs for IoT, explainable AI for security, and explainable DL-based IDSs. The overview of relevant concepts is presented in Section 3. Section 4 describes the methodology of the study, Section 5 presents the findings of the result questions, discusses the interpretation of the findings, and their implications for research and practice. Section 6 contains the future research directions. Lastly, Section 7 concludes the study.

## 2. Related Works

In this section, we review some survey articles that have been published on IDSs for IoT networks, explainable AI for security, as well as explainable DL-based IDSs, highlighting their major contributions and drawbacks.

### 2.1. Reviews on IDSs for IoT

Several review studies have been carried out on the deployment of network-based intrusion detection systems for IoT networks. For instance, Abdulkareem et al. [33] present a comprehensive examination of network intrusion detection (NID) methodologies, particularly in the context of IoT. The study assesses both traditional and IoT-specific NID approaches, highlighting their similarities and differences. The analysis categorizes datasets into conventional and IoT-specific types, evaluates various supervised machine learning classifiers, and emphasizes their effectiveness in detecting intrusions within IoT networks. The study underscores a notable shift from traditional datasets to those that better reflect modern network configurations, indicating a growing focus on IoT in NID research. Emerging trends are identified, along with suggestions for future research directions.

Heidari and Jabraeil [20] present a systematic review of intrusion detection systems specifically designed for IoT applications. It assesses the strengths and the drawbacks of current IDS methodologies and suggests future research directions to enhance their implementation. The review emphasizes the complexity and diversity of attacks in IoT environments, noting that many existing detection solutions fall short in addressing all potential threats. Additionally, the study highlights a preference for simulation-based approaches over real-world implementations. The paper concludes by identifying unresolved challenges in the design and implementation of IDSs for IoT, offering recommendations for future research and development aimed at improving detection accuracy and operational effectiveness in real-world IoT scenarios.

Liao et al. [34] address the increasing security challenges within the IoT ecosystem and examine how deep learning can enhance IDSs. It underscores the necessity for effective IDSs in IoT environments due to the massive volume of data generated by IoT devices. The study focuses on the advantages of deep learning over traditional methods by providing a comparative analysis of various models, including Convolutional Neural Networks (CNNs), Generative Adversarial Networks (GANs), and Long Short-Term Memory (LSTM) networks. The paper emphasizes the significance of dataset selection for effective model training and discusses preprocessing and feature extraction techniques that impact model performance. It emphasizes that deep learning models can significantly enhance detection efficiency and accuracy by automatically identifying patterns and anomalies within network behavior, thus reducing reliance on manual feature engineering. In a similar study, Khan et al. [35] surveyed IDSs using deep learning techniques that focus on IoT networks. They evaluate various IDSs and assesses their effectiveness using publicly available network-based datasets. The research identifies key challenges related to network security and privacy and offers potential solutions. The investigation analyzes deep learning models like Autoencoders, Gated Recurrent Units (GRUs), and CNNs for anomaly detection. The paper shows that deep learning techniques enhance IDS performance by detecting complex malicious behaviors more effectively than traditional methods. The study also addresses challenges such as noisy data, imbalanced datasets, and the need for continuous updates to counter evolving attacks. Proposed solutions include feature normalization, adversarial sample generation, and algorithmic improvements through transfer learning and reinforcement learning.

Similarly, Asharf et al. [36] present a comprehensive review of IDSs employing ML and DL techniques in the context of IoT security. The survey addresses six key areas: IoT architectures and technologies, IoT threats and attack types, IDS architectures and design, ML and DL techniques, available datasets, and future research directions. The review categorizes techniques based on their applications in detecting intrusions while emphasizing the necessity for robust security measures, considering the inherent constraints of IoT devices, such as limited power and bandwidth. It highlights that traditional IDS methods often fall short in IoT environments due to unique characteristics, including energy limitations and the diversity of devices. This underscores the importance of adapting intrusion detection strategies to better meet the specific needs of IoT systems.

### 2.2. Surveys on Explainable AI for Security

Explainable Artificial Intelligence is essential in cybersecurity. It renders complex ML and DL models more interpretable. These models find applications in systems like NIDS and IoT security. XAI increases transparency, understandability, and trust. This allows security analysts to understand AI decisions and avert risks successfully [37]. With increasingly complex cyber-attacks, particularly in IoT settings, the need for explainable models to analyze alerts and reduce false positives has grown [38]. Existing reviews on XAI in cybersecurity provide essential insights into its applications, focusing on NIDS and IoT-specific concerns, but lack insights on performance metrics, necessitating a study to fill the identified gaps.

A comprehensive review conducted by Rjoub et al. [39] examines XAI techniques like SHAP (SHapley Additive exPlanations) and LIME (Local Interpretable Model-agnostic Explanations) applied to NIDS, malware detection, and phishing prevention. It emphasizes how SHAP provides feature importance for NIDS alerts with reduced false positives. The study systematically classified cybersecurity threats and assessed the applicability of XAI methods to these scenarios, while also discussing the challenges, limitations, and potential future research directions within this interdisciplinary field. While the survey indicates the way forward in closing the gap between complex models and human analysts using XAI, it lacks a focused performance analysis related to the effectiveness and efficiency of XAI techniques in NIDS. This is crucial for demonstrating how well these techniques can operate under the various conditions and constraints typically encountered in real-world cybersecurity environments.

Charmet et al. [40] provide a review on the intersection of XAI and cybersecurity, addressing the challenges of making AI models interpretable for human users, particularly security operators who deal with numerous alerts daily. It emphasizes that while AI and machine learning have become essential for developing cybersecurity solutions, such as malware detection and intrusion prevention, these systems often fail to justify their results, leading to potential misinterpretations and alert fatigue. The authors investigate the applications of XAI in cybersecurity, focusing on its role in enhancing decision-making processes for security events, and highlight common vulnerabilities in existing AI approaches. Furthermore, the paper explores the security implications of XAI systems themselves, discussing potential attacks on XAI methodologies and proposing areas for further research. Ultimately, the survey underscores the need for explainable and secure AI solutions to address the complexities of real-world cybersecurity, but it lacks any form of evaluation criteria for XAI implementations in this field.

In another survey, Zhang et al. [41] review XAI applications in cybersecurity, highlighting the need for transparency and interpretability in AI-driven security measures. As ML and DL techniques are used to detect cyber threats, many systems operate as “black boxes,” hindering user understanding and trust. This opacity can undermine confidence in AI models, especially as cyber-attacks grow in complexity. The study examines XAI literature in various applications, including intrusion detection, malware identification, and spam filtering, while addressing associated challenges and motivations. It evaluates state-of-the-art XAI solutions and proposes a framework for categorizing these applications. However, the study lacks a focus on the unique challenges posed by IoT environments, such as resource constraints, heterogeneous devices, and dynamic network traffic.

Srivastava et al. [42] review XAI and its applications in cybersecurity, addressing the challenges of traditional AI models that lack transparency and interpretability. The paper begins by outlining basic concepts of cybersecurity and detailing various cyber threats that require advanced threat detection systems. It emphasizes that AI and machine learning approaches yield results that are hard to interpret, making it difficult for cybersecurity professionals to understand the reasoning behind specific decisions, which can erode trust and effectiveness when timely decision-making is critical. The review highlights XAI’s potential to address these challenges by providing clearer insights into cyber threat detection and response, enhancing cybersecurity frameworks with interpretable and justified results. However, while the study mentions the need for XAI in IoT contexts, it does not explore the operational effectiveness of these frameworks when deployed in IoT environments, potentially leaving a gap in understanding their practical implications in real-world scenarios.

### 2.3. Studies on Explainable DL-Based IDSs

A lot of studies have been conducted on explainable deep learning-based intrusion detection systems. These studies focus on the integration of explainable artificial intelligence methods in enhancing the interpretability and effectiveness of IDSs. For instance, Mohale and Obagbuwa [43] investigate the integration of XAI techniques within IDSs to enhance transparency and interpretability in cybersecurity. The study highlights the increasing need for cybersecurity systems that not only deliver high accuracy in detecting sophisticated cyber threats but also provide clear, interpretable insights that security analysts can readily understand and trust. The review focuses on popular XAI techniques such as SHAP, LIME, decision trees, and hybrid models, discussing their benefits and limitations in IDS applications. The authors underscore that while model-agnostic explanations improve interpretability, they face challenges like computational inefficiency and difficulties in real-time deployment.

Samed and Sagiroglu [44] examine the role and importance of explainable artificial intelligence within IDSs in cybersecurity. As AI technologies, particularly deep learning models, grow increasingly complex and opaque, the need for transparency and interpretability in security applications becomes essential. The paper explores various XAI methods applied to IDSs, emphasizing how explainability enhances trust, enables better understanding of detection decisions, and assists cybersecurity professionals in handling false positives, false negatives, and attack analyses. It also discusses the increasing research attention given to XAI-based IDSs and provides an overview of current explainability techniques, datasets, and evaluation criteria used in this area. Furthermore, the study outlines critical challenges in integrating XAI with IDSs, such as vulnerabilities to attacks that exploit the transparency of models, inherent biases in training data that can mislead explanations, and difficulties in designing explanations that are both accurate and meaningful for cybersecurity experts.

Mendes and Rios [45] present a systematic review of XAI techniques in cybersecurity, addressing the challenges of black-box AI models, like deep neural networks, which are often opaque and difficult to interpret. The authors emphasize that explainability in AI is essential for building user trust and for helping researchers and security experts understand, debug, and enhance AI-based cybersecurity solutions. The study identifies several common XAI techniques used in cybersecurity, with SHAP and LIME being the most prominent due to their availability in open-source frameworks and model-agnostic nature. Other notable techniques include decision trees, example-based explanations, adversarial methods, micro-aggregation with decision trees, and autoencoder-based approaches.

Ramya et al. [46] review how XAI is advancing cybersecurity. They discuss the integration of AI techniques, such as machine learning and neural networks, in threat detection, malware identification, intrusion detection, and botnet detection. The authors highlight the importance of transparency and interpretability in AI systems to build trust among cybersecurity professionals. They explore recent developments in XAI tools like LIME and SHAP, which explain AI decisions. The paper also addresses ethical, regulatory, and technical challenges in deploying XAI in cybersecurity, including data privacy, bias, and computational complexity. Finally, it advocates for future research and standardization to enhance AI-based cybersecurity solutions. Similarly, Pawlicki et al. [47] explores the application of XAI in deep learning and cybersecurity, emphasizing its importance for transparency, trust, and performance in security systems like NIDS. It highlights cybersecurity’s critical role in protecting infrastructure and discusses challenges posed by opaque, black-box ML models that hinder understanding and trust. The authors review XAI techniques, distinguishing between local and global explanation methods, and conduct a systematic mapping study to identify research trends and future directions. Key research opportunities include developing user-centered, context-aware explanations, integrating interactive and hybrid approaches, addressing biases, and establishing quantitative evaluation metrics. The study emphasizes tackling ethical considerations and vulnerabilities linked to explainability techniques, balancing model accuracy with transparency to foster trust and effective decision-making, and presents a roadmap that prioritizes human needs, ethics, and technical robustness for trustworthy, interpretable AI systems. Table 1 provides a summary of all related works highlighting their key contributions and drawbacks.

### 2.4. Summary of Related Work and Research Gaps

The reviewed literature indicates that early studies established deep learning as a transformative approach for IoT intrusion detection, primarily aimed at enhancing detection accuracy and addressing the limitations of traditional systems. However, the emergence of the black-box problem prompted further research advocating for explainable AI (XAI) in cybersecurity. This body of work has cataloged techniques such as SHAP and LIME, emphasizing their importance for building trust, assisting analysts, and facilitating debugging. Recent integrative studies have begun to merge these two areas, showcasing functional explainable deep learning-based intrusion detection systems (IDSs) while also recognizing emerging challenges, such as computational overhead. This evolution has taken place separately, as one path emphasizes optimizing detection performance, another focuses on interpretability, and a third aims to combine the two descriptively. Consequently, there is a significant analytical gap at the intersection of these research paths. This gap highlights the absence of a unified, systematic examination of the essential performance–efficiency–explainability trilemma, which is critical for the real-world applicability of resource-constrained IoT environments. Thus, this study addresses the underexplored gap at the intersection of performance, efficiency and explainability by analyzing the severe trade-offs between high explainability and efficiency, which can make IoT deployment infeasible, and highlighting the lack of reported computational metrics as a major barrier to real-world progress. Additionally, it exposes the explanation validation gap, demonstrating that current work lacks rigorous assessment of explanation quality and utility, and offers frameworks to advance from generating explanations to empirically proving their value for security operators.

## 3. Overview of Relevant Concepts

Here, we present some commonly used DL models for IDSs, as well as explainability techniques used for IDSs. Furthermore, we present an overview of IoT technologies and their communication protocols, and finally, we describe some key performance metrics relevant to this study.

### 3.1. Deep Learning Models for IDSs

Deep learning models have been deployed with IDSs for better model accuracy, particularly in IoT environments, due to these models’ ability to identify patterns from large datasets. Moreover, DL models can automatically model a complex feature set from sample data. In what follows, we briefly describe some common DL architectures used in network intrusion detection, focusing on their strengths and drawbacks in the context of IoT network traffic analysis.

#### 3.1.1. Convolutional Neural Networks (CNNs)

The CNN, as a discriminative DL algorithm, is designed to reduce the amount of data inputs required for a conventional artificial neural network through the use of equivalent representation, sparse interaction, and sharing of parameters [36]. The architectural distinction of CNNs from traditional artificial neural networks is the substitution of matrix multiplication with convolutional computation, a modification that confers enhanced data processing performance [34]. The structure of a CNN is a three-layer architecture, which is typically composed of a convolutional layer, a pooling layer, and a fully connected layer [34,48]. The convolutional layers detect local features through convolution operations, the pooling layers help reduce dimensionality and select prominent features, and the fully connected layers combine these features to classify or predict the output [49]. These models are capable of automatic feature learning and extraction, which reduces the reliance on manual feature engineering, though it requires high computational power.

In the context of IDSs, CNNs have demonstrated remarkable success in analyzing network traffic patterns [50]. The ability to automatically learn hierarchical representations of data, starting with low-level features like packet headers and progressing to high-level patterns indicative of malicious activity, makes CNNs particularly well-suited for identifying subtle anomalies in IoT environments [51]. CNNs excel at handling the high dimensionality and complex temporal relationships within IoT network traffic, where numerous devices and protocols generate a vast volume of data [50]. This inherent capacity for feature extraction allows for the detection of sophisticated attacks that might elude traditional signature-based systems. Furthermore, the robustness of CNNs to noisy and incomplete data, which is often common in IoT deployments, is a significant advantage [52].

However, deploying CNNs for security on resource-constrained IoT devices is highly challenging. This is partially overcome through a distributed architecture: a lighter version of the deep neural network, trained on a subset of vital output classes and run on the device, while the full algorithm training is then offloaded to the cloud. Furthermore, the complex architecture of CNNs can make them less transparent compared to traditional methods, potentially obscuring the reasoning behind intrusion detection.

#### 3.1.2. Recurrent Neural Networks (RNNs)

RNNs, as neural network architectures, are designed to handle sequential data, such as network traffic over time. They achieve this by cycling information through the network via shared weights, allowing the model to maintain contextual memory of previous inputs and apply this knowledge to current data [34]. RNNs are effective for tasks involving time series or other sequential patterns, making them suitable for intrusion detection in network traffic analysis. However, RNNs face significant challenges related to training, notably the issues of gradient explosion and vanishing gradients, which impair their ability to learn long-range dependencies in data [34,53]. Due to the large time span, the network cannot remember information for a long time, which hampers its ability to effectively learn and retain long-term dependencies.

To solve this problem, RNNs model data as sequential and time-dependent, capturing temporal dependencies by maintaining a hidden state that preserves information across input sequences. The output generated by each hidden unit is determined not only by the current input at a specific timestep but also by the output produced by that same hidden unit during the previous timestep. This is made possible through a feedback loop within each hidden unit, which takes the unit’s own previous output and feeds it back into itself. As a result, this feedback mechanism enables the network to retain and carry forward information over time, allowing it to understand and model sequential data by associating information from earlier timesteps with future ones [53]. Some more advanced variations of RNNs that address the vanishing gradient problem include long short-term memory (LSTM) [54] and gated recurrent unit (GRU) [55].

LSTM networks are an advanced form of recurrent neural networks, RNNs, designed to handle sequential data, particularly where long-term dependencies are involved. Unlike traditional RNNs, LSTMs maintain a cell state that allows them to remember information over extended sequences [54]. This memory is regulated by input, forget, and output gates that control the flow of information into and out of the cell state [25]. Some specialized architectures designed to improve or adapt the basic LSTM models are stacked LSTM, bidirectional LSTM, convolutional LSTM, and peephole LSTM, each with a distinct purpose [56].

The GRU is a lightweight version of LSTM specifically tailored to manage sequential data, particularly when intricate, long-term dependencies are present within the information stream. Unlike conventional RNN architectures, the GRU leverages a streamlined gating mechanism. This streamlined approach, while simplifying the computational burden compared to more complex architectures like LSTMs, nonetheless achieves comparable performance in learning intricate long-range dependencies [55]. This streamlined performance is achieved through the strategic use of update and reset gates, which meticulously govern the flow of information within the network’s internal processing [57]. The major difference between LSTMs and GRUs lies in their gating mechanisms. LSTMs have a more complex, three-gate structure: input, output, and forget gates, allowing for more granular control over information flow. On the other hand, GRUs use a simpler two-gate structure, which includes update and reset gates. This simpler structure results in a reduced computational cost for GRUs, making them potentially faster and more memory-efficient [58].

With regard to IDSs, the three DL models, RNN, LSTM, and GRU, have been widely used for their ability to analyze network traffic effectively [59,60]. In IoT network traffic analysis for IDSs, RNNs can capture sequential patterns but struggle with long-term dependencies due to vanishing gradients. LSTMs overcome this limitation with gating mechanisms that enable them to model complex, long-term attack patterns effectively, making them well-suited for detecting sophisticated threats. GRUs, offering a simpler and faster alternative with fewer parameters, are advantageous for real-time monitoring in IoT resource-constrained environments, though they may be less effective than LSTMs in capturing very long-term dependencies. Ultimately, the choice among these models depends on specific deployment needs, balancing factors like processing speed, resource availability, and the complexity of network threats.

#### 3.1.3. Autoencoder

Autoencoders, as a specialized neural network, are designed to learn efficient, compressed representations of unlabeled data, primarily for tasks such as dimensionality reduction and feature learning. The core architecture consists of two main components: the encoder, which maps the input data to a lower-dimensional latent space, and the decoder, which reconstructs the input from this latent representation. This process forces the network to prioritize and capture the most salient features of the data while filtering out noise and less relevant information. Autoencoders can be trained using techniques like stochastic gradient descent and backpropagation, allowing them to learn complex, non-linear data representations that preserve important patterns within the data [61]. Some variations of autoencoder networks are variational, adversarial, and convolutional autoencoders. Each architecture introduces specific enhancements, such as improved generative capabilities, robustness to noise, or tailored processing of visual data [62].

In relation to NIDS, autoencoders are effective in detecting unknown types of attacks (i.e., zero-day attacks) and can also eradicate the problem of an arduous labeling task [63]. Their unsupervised learning capability allows them to model normal network traffic patterns, enabling the detection of anomalies that deviate from learned behaviors. This makes autoencoders particularly useful in IoT network traffic analysis, where new and evolving threats frequently emerge, and labeled datasets are often scarce or incomplete [64]. Additionally, their ability to compress high-dimensional data into lower-dimensional representations helps in efficiently processing the massive volume of traffic generated by IoT devices, facilitating real-time intrusion detection. However, autoencoders also have limitations when applied to IoT network traffic analysis. One significant weakness is their tendency to produce false positives, especially when normal traffic patterns are highly variable or exhibit seasonal variations, which can lead to alert fatigue and reduced trust in the system [63].

#### 3.1.4. Transformers

The transformer, as a sequence-to-sequence model, employs an encoder–decoder structure to transform input symbolic sequences into continuous vector representations, which are then used to generate output sequences. Unlike traditional recurrent models, the transformer relies solely on self-attention mechanisms that enable it to weigh different parts of the input data differently, capturing contextual relationships efficiently [65]. The encoder processes the input sequence through multiple layers to generate rich, high-dimensional features, which the decoder then uses to produce the output sequence [66]. This attention mechanism operates via scaled dot-product attention, where query, key, and value matrices compute attention scores that determine the importance of each element relative to others, facilitating parallel processing and reducing the dependence on sequential data [67].

In the context of intrusion detection, the transformer’s ability to handle complex, ordered data makes it highly suitable for processing network traffic features. Its self-attention mechanism allows the model to learn varying weights for different features, capturing their significance and interactions effectively. The model’s architecture enables it to extract meaningful representation from high-dimensional raw data, balancing feature reduction with the retention of critical information [66,68]. This makes the transformer a robust foundation for tasks like anomaly detection in network traffic, where understanding contextual relationships and feature importance is crucial for accurate classification and detection of malicious activities. Furthermore, transformer-based models can be pre-trained on large datasets and fine-tuned with smaller datasets, which is beneficial when dealing with limited IoT traffic data [69]. This allows for improved efficiency and security in IoT networks by classifying traffic effectively. However, transformer models can require significant computational resources and large amounts of data for effective training. Furthermore, the interpretability of these models can be a challenge, making it difficult to understand the reasoning behind their classifications [70].

### 3.2. Explainability Frameworks and XAI Techniques

**Figure 1:**
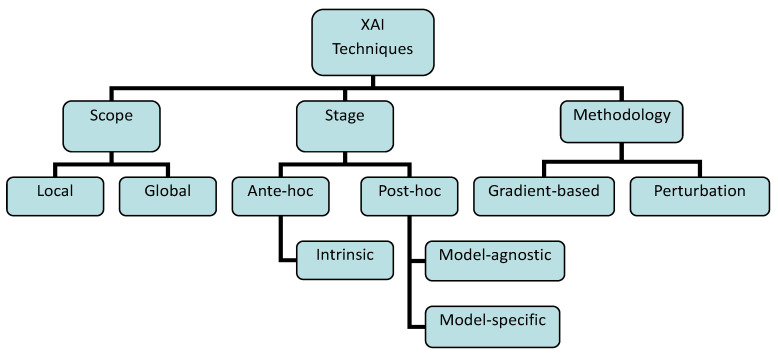
Overview of XAI techniques classification.

In this section, we present an overview of the different categories of XAI techniques and their applicability to deep learning models. Explainability techniques can be categorized as either local or global, ante hoc or post hoc, and gradient-based or perturbation-based [71,72], as shown in Figure 1.

#### 3.2.1. Local vs. Global XAI Techniques

The scope of explanation is one way to categorize explainable AI techniques. It defines the extent of an explanation produced by an XAI method. An explanation may either cover the entire model or focus on specific input instances. Based on this scope, explanations are typically classified as either local, detailing individual predictions, or global, providing an overview of the entire model [73].

Local explanation methods aim to provide insights into the decision-making process of an AI model for individual predictions. These techniques focus on explaining why the model produced a specific output for a particular input, helping users understand the factors that influenced that decision [74]. Common approaches include feature attribution methods like LIME (Local Interpretable Model-agnostic Explanations) [75] and SHAP (SHapley Additive exPlanations) [76], which identify the most influential features contributing to a single prediction. These explanations are especially useful in high-stakes applications where understanding the rationale behind a specific outcome is crucial for trust, validation, and addressing potential biases [30]. Local explanation techniques are particularly valuable for deep learning models because they help demystify the decision-making process at the level of individual predictions, which are often opaque due to the complex, layered structure of neural networks.

Global explanation methods, in contrast, aim to provide a comprehensive understanding of the entire model’s behavior across all inputs. These techniques describe how the model makes decisions in general, revealing the overall patterns, feature importance, and decision boundaries [71]. Some common techniques include feature importance rankings, partial dependence plots, and model simplification methods like rule extraction or surrogate models. Such explanations are valuable for model validation and gaining insights into the underlying data relationships. They help stakeholders understand the general logic of the model rather than focusing on individual predictions, fostering transparency and trust in the AI system [30]. While deep learning models are inherently complex, global explanations help stakeholders grasp the general patterns learned by the network, facilitating model debugging and improved transparency.

#### 3.2.2. Ante-Hoc vs. Post-Hoc XAI Techniques

Another approach to classifying XAI techniques is based on whether a model is inherently explained (ante hoc) or explained by using XAI toolboxes that analyze the model after training (post hoc). Ante hoc and post hoc explanation techniques are two distinct approaches for explaining the inner workings of AI systems, differentiated primarily by the stage at which they are applied [72,77]. Ante hoc techniques are utilized during the training and development phases of an AI system to enhance transparency and understanding of the model. In contrast, post hoc techniques are implemented after the AI models have been trained and deployed, serving to clarify the model’s predictions or decision-making processes for users. Post hoc explainability is particularly relevant for models that are not easily interpretable through ante-hoc methods.

- Model-Agnostic vs. Model-Specific XAI Techniques

The post hoc explanation can be classified into two categories: model-agnostic and model-specific. The model-specific methods focus on exploring the inner workings of a model, such as examining its architecture and intermediate representations to interpret its decisions. In contrast, model-agnostic techniques analyze features, their relationships with outputs, and the underlying data distribution, regardless of the model’s internal design [71].

Model-agnostic techniques provide explanations for all AI models, irrespective of their internal workings or design. They are particularly valuable for complex models that resist explanation through traditional methods. This flexibility extends to the model itself, the explanations produced, and the representations used, making them applicable to a broad spectrum of models [78]. Model-agnostic techniques are based on the principle of decoupling explanation from the specifics of the model’s architecture. Instead of trying to understand the model’s internal logic, the technique focuses on observing the model’s input–output behavior. This allows for the analysis of models without requiring access to their internal computations, making them invaluable for black-box models. These methods are not limited to a particular type of model or explanation format. This inherent flexibility is particularly important in the context of deep learning models used in IoT networks, where models often exhibit complex, non-linear behavior and their architecture is often opaque [72]. Model-agnostic methods can provide valuable insights into how these models function, potentially revealing biases, vulnerabilities, or areas for improvement without needing to delve into the model’s intricate inner workings. This is critical in IoT networks where understanding model behavior is paramount for safety, reliability, and security.

Model-specific techniques analyze a particular AI model’s inner workings to understand its predictions or decisions. These techniques, tailored to a specific model’s architecture, may not be transferable to other models. However, they offer valuable insights into the model’s decision-making process. For instance, model-specific approaches can illuminate how neural networks, random forests, or support vector machine function [72,79]. Model-specific techniques rely on leveraging the model’s architecture. They dissect the internal processes, such as neuron activations in neural networks, feature importance in random forests, or decision boundaries in support vector machines. This direct examination into the model’s mechanics allows for a deep understanding of how the model arrives at its outputs. However, this focused approach also limits its applicability to only the specific model for which it was designed [80]. In the context of IoT networks, model-specific techniques can be valuable for understanding how a particular deep learning model classifies sensor data, predicts device failures, or optimizes resource allocation, but their utility is restricted to that particular model architecture.

#### 3.2.3. Gradient-Based vs. Perturbation-Based Techniques

Another criterion for categorizing XAI techniques is to consider their reliance on the model’s training process. Gradient-based methods, often used with deep learning models, leverage the gradients of the loss function to understand how changes in input features affect the model’s predictions. Perturbation-based methods, on the other hand, often operate independently of the model’s internal structure, instead focusing on making small changes to the input data and observing the model’s response to those changes [81].

Gradient-based techniques describe XAI methods that explain models by leveraging the gradients of the model’s output with respect to its input features. These methods analyze how small changes in the input affect the output, and the magnitude of the gradient indicates the influence of each feature [82]. The techniques used often involve calculating the gradient of the model’s output with respect to each input feature, and then using this gradient information to assess the importance of each feature to the model’s prediction. Methods like Integrated Gradients [83] and Saliency Maps [84] are common examples. These techniques are often computationally efficient, relying on the model’s internal representation through backpropagation [85]. Gradient-based techniques quantify the sensitivity of the model’s prediction to changes in input values, with larger gradients indicating greater feature importance. This approach is suitable for deep learning models because it leverages the inherent gradient information available through backpropagation.

On the other hand, perturbation-based methods explain models by systematically modifying or masking parts of the input data to observe changes in the model’s output. By intentionally perturbing inputs, such as through occlusion, feature substitution, or random sampling, these methods assess the sensitivity of the model’s predictions to different features without relying on internal gradients [86]. This makes them highly applicable to deep learning models, where internal structures can be complex and opaque, as well as to IoT networks, which often generate heterogeneous and high-dimensional data [87]. Methods like LIME [75] and SHAP [76] are common examples, generating feature importance maps by analyzing the impact of input perturbations [81]. These techniques are often model-agnostic and provide human-interpretable explanations based on how input modifications alter predictions.

### 3.3. Performance Analysis Metrics

Generally, to evaluate the performance of NIDS, there are sets of traditionally used detection accuracy metrics, which in most cases do not give a holistic evaluation metric, especially for XAI-enhanced DL-based NIDS [88,89]. Evaluating the performance of XAI-enhanced DL-based NIDS for IoT networks requires a multi-dimensional approach that extends beyond the commonly used accuracy metrics, considering factors such as interpretability, robustness, computational efficiency, and the system’s ability to adapt to evolving threats, to ensure a comprehensive understanding of its effectiveness and practical viability. In what follows, we categorize the performance metrics and briefly discuss them.

#### 3.3.1. Detection Accuracy Metrics

Detection accuracy metrics are used in NIDS to assess how well an intrusion system identifies and classifies network intrusions by measuring the proportion of correctly identified malicious activities relative to the total number of intrusions [90]. Classification performance in NIDS involves evaluating how accurately the system distinguishes between normal and malicious traffic, characterized by metrics such as true positives (TPs—correctly detected intrusions), true negatives (TNs—correctly identified normal traffic), false positives (FPs—normal traffic incorrectly flagged as malicious), and false negatives (FNs—missed intrusions), which collectively indicate the system’s detection capability and reliability. Some commonly used detection accuracy metrics are described in Table 2.

#### 3.3.2. Computational Overhead Metrics

Assessing the resource demands of an NIDS is essential, particularly within IoT environments where devices often have limited processing power, memory, and energy reserves. Key metrics such as CPU utilization, memory consumption, power usage, and system latency provide crucial insights into how efficiently an NIDS can operate without overwhelming the constrained resources of IoT devices and edge infrastructure [91]. These metrics are vital for evaluating the deployability and scalability of intrusion detection systems, ensuring they can function effectively in real time while maintaining minimal impact on device performance and energy consumption. The following metrics used for quantifying computational overhead are described in Table 3.

#### 3.3.3. Explainability Quality Metrics

Evaluating the usefulness and impact of explanations generated by XAI for IDSs, especially within the constraints of IoT environments, requires a more comprehensive approach than simply examining technical metrics [92]. While quantitative measures like fidelity, sparsity, and stability, as shown in Table 4, provide insight into the technical aspects of these explanations, they fall short of capturing the crucial human element: how meaningful, understandable, and ultimately actionable the explanations are for human users [93,94] such as security professionals.

To evaluate the effectiveness and efficiency of an XAI-enhanced NIDS for an IoT environment, it is essential to try to achieve a balance across all these categories of performance metrics rather than focusing on just one category. This approach will provide a robust, deployable, and scalable system with useful explanations that can be easily understood by security professionals for decision-making.

## 4. Research Methodology

Utilizing the preferred reporting items for systematic reviews and meta-analysis (PRISMA) methodology proposed in [95], we present a comprehensive protocol for the systematic review of the literature. This includes the research questions, search strategy, study selection criteria, data extraction process, and the data synthesis process.

### 4.1. Research Questions

The research questions (RQs) guiding this study are categorized into four areas: XAI technique trade-off, model comparison, XAI evaluation, as well as bottleneck and mitigation. For ease of understanding, the RQs are divided into sub-questions as presented in Table 5.

### 4.2. Search Strategy

**Figure 2:**
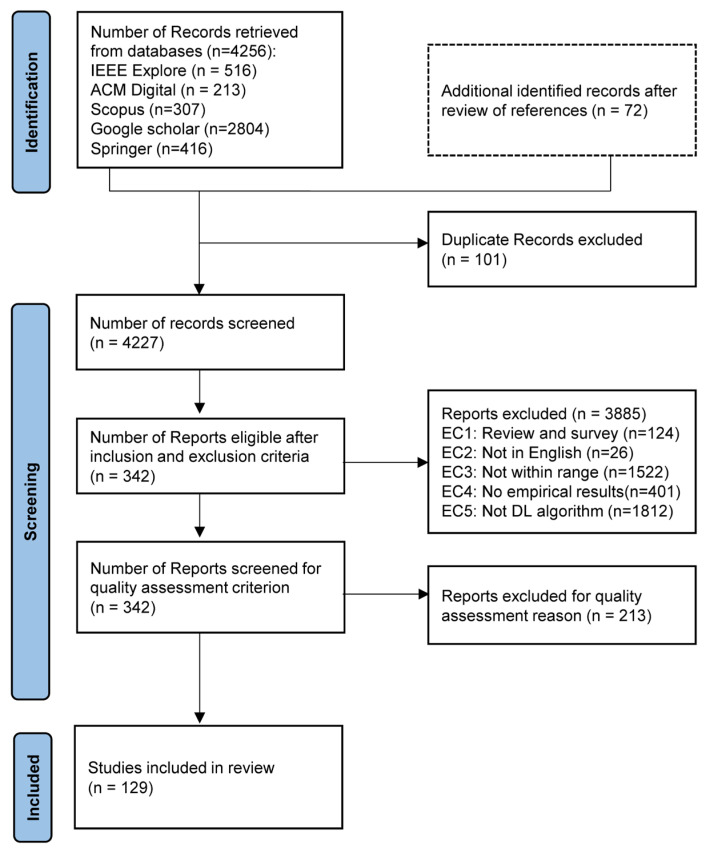
PRISMA flow diagram showing the process for the selection of the included studies (initial records: *n* = 4328; final included studies: *n* = 129).

To conduct a thorough systematic search of the literature, we performed a comprehensive search across five major academic repositories: IEEE Xplore, ACM Digital Library, Scopus, Google Scholar, and Springer. The search focused on recent literature from 2018 through May 2025. To ensure an effective search strategy, the initial step involved selecting appropriate keywords. The search terms used included: “explainable AI,” “XAI,” “Interpretable AI,” “intrusion detection system,” “IDS,” “network security,” “IoT,” “Internet of Things,” “performance,” and “evaluation.” These keywords were combined using Boolean operators (AND, OR) to formulate precise search queries. Additionally, synonyms of these terms were used on the repositories, as presented in Table 6, except for Scopus which does not support wildcards, to broaden the search scope. As a result, a total of 4328 articles were retrieved. The practical management of the large search space, particularly from Google Scholar, and the subsequent removal of duplicates was achieved through a structured process. To handle the broad yield from Google Scholar, the search was limited to the first 2800–3000 relevance-sorted results to ensure a manageable and pertinent initial dataset. Deduplication was then performed semi-automatically by using fuzzy matching and manual inspection to identify duplicates based on highly similar titles and matching author/year metadata. This rigorous process successfully filtered the initial 4328 records down to 4227 unique articles for formal screening. Figure 2 gives a breakdown of the process for the final selection of included studies using PRISMA guidelines.

### 4.3. Inclusion/Exclusion Criteria

Inclusion and exclusion criteria were established to identify and select the most relevant articles for the study. The inclusion and exclusion criteria (IC and EC) presented in Table 7 were systematically established to ensure relevance, methodological rigor, and alignment with the objectives of this review, following PRISMA guidelines for systematic literature reviews. The inclusion criteria were derived directly from the defined research questions and were designed to capture studies that (i) address intrusion or anomaly detection, (ii) employ deep learning techniques only, (iii) explicitly integrate explainability mechanisms, and (iv) focus on IoT networks or IoT-generated traffic, thereby ensuring domain specificity. The requirement for empirical results or analytical discussion was imposed to enable meaningful performance-oriented synthesis across studies. Conversely, exclusion criteria were applied to avoid duplication, ensure quality, and maintain comparability across the selected literature, as utilized in Figure 2 and depicted in Table 7. Together, these criteria provide a transparent and reproducible filtering process that supports the validity and focus of the systematic review.

### 4.4. Quality Assessment and Critical Appraisal

After the collected articles were filtered using the inclusion and exclusion criteria, 342 papers related to our study interest remained for quality assessment. To ensure methodological rigor beyond basic relevance screening, we implemented a structured multi-criteria quality assessment, presented in Table 8. All 342 studies underwent independent evaluation using a standardized appraisal instrument to determine their suitability for inclusion in the final analysis.

#### 4.4.1. Quality Assessment Instrument

We developed a domain-specific quality assessment checklist comprising six criteria organized into four dimensions: methodological rigor, reporting quality, relevance, and validity, shown in Table 8. Each criterion was scored on a three-point scale: 2 (Fully Met), 1 (Partially Met), or 0 (Not Met). Studies could achieve a maximum score of 12 points. This multi-dimensional approach enables granular and reproducible judgments of study quality, moving beyond binary relevance screening to assess the robustness, transparency, and reliability of each study’s contribution to our research questions.

#### 4.4.2. Quality Assessment Implementation and Results

Based on total quality scores, studies were classified into three tiers: (i) High Quality (10–12 points): Studies demonstrating rigorous methodology, comprehensive reporting, and strong alignment with research questions. 51.9% of the included studies (67 studies) fall under the high-quality category. These form the primary evidence base for synthesis. (ii) Moderate Quality (6–9 points): Studies with acceptable methodology but notable reporting gaps or partial alignment with RQs. This category accounted for 48.1% of included studies with 62 studies. Included but flagged for sensitivity analysis. (iii) Low Quality (0–5 points): Studies with significant methodological concerns, minimal reporting, or unclear relevance. Excluded from final analysis (*n* = 213). We established a minimum threshold of 6 points (50% of maximum score) to ensure that included studies met at least moderate standards across all four dimensions. This threshold balances inclusivity with quality assurance, excluding only those studies with pervasive methodological or reporting deficiencies.

### 4.5. Data Synthesis

For the synthesis of evidence, we employed a mixed-methods approach integrating both quantitative and qualitative data extracted from the included studies. The synthesis was guided by the four research questions. Our aim with this approach was to identify overarching trends and gaps across the body of literature. For quantitative aspects, specifically addressing how various XAI techniques and deep learning architectures impact detection accuracy and computational overhead, as well as the evaluation of XAI techniques after deployment (RQ1, RQ2 & RQ3), a comparative analysis of reported metrics was performed. This involves aggregating and categorizing data to highlight trends, trade-offs, and performance variations, complemented by qualitative interpretations of observed differences.

For the qualitative elements, focusing on identifying primary deployment bottlenecks and mitigation strategies (RQ4), qualitative thematic analysis was applied. The overall synthesis was concluded by integrating these findings to provide comprehensive answers to the research questions, highlight research gaps, and propose future directions for the field. The information extracted from all the 129 included studies is available on GitHub “https://github.com/TaiwoBlessyn/Performance-Analysis-XDL-IDS-IoT (accessed on 2 January 2026)”.

## 5. Results

In this section, we present the results of the study and provide an in-depth analysis of the findings based on each RQ. Furthermore, we synthesize the data to identify patterns, highlight significant trends, and discuss the implications of these findings.

### 5.1. Overview of Included Studies

**Figure 3:**
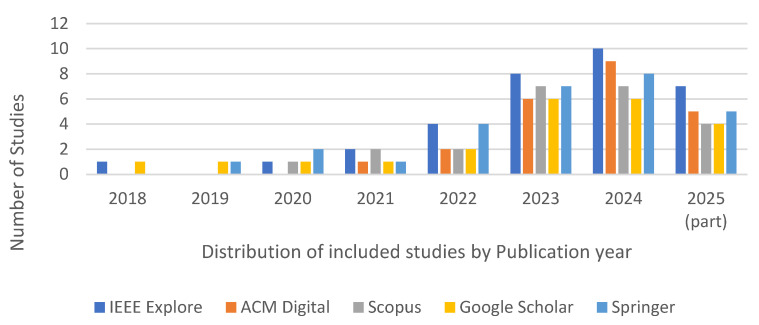
Distribution of included studies by publication year and various academic repositories (*n* = 129 included studies). Studies were categorized based on the source database from which they were retrieved.

A descriptive summary of the selected studies for the systematic review is given below. Based on the comprehensive search, we identified a total of 129 studies that met our predefined inclusion criteria. The distribution of these studies by publication year and academic repository is presented in Figure 3, and their characteristics are summarized below, providing a foundational context for the findings that follow. The field of explainable deep learning for IoT intrusion detection systems has experienced a notable surge in research activity over the past few years. As shown in Figure 3, the number of relevant publications has steadily increased, reflecting a growing interest in addressing the explainability of “black-box” models used in critical security applications. This trend underscores the significance of this research domain.

**Figure 4:**
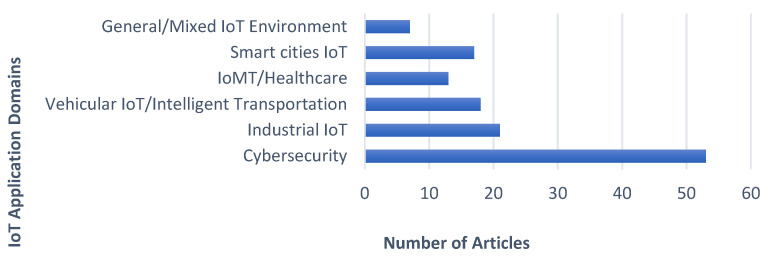
Distribution of included studies across IoT Application Domains (*n* = 129 included studies). Studies were classified into six domain categories based on the stated application context in each paper’s introduction or methodology sections.

The included studies span a wide array of IoT application domains, as presented in Figure 4, indicating the broad applicability of explainable IDSs. The most frequently studied domain is cybersecurity, accounting for 41% of the publications. Other significant domains include: Industrial IoT (IIoT), this involves studies focusing on securing critical infrastructure and industrial control systems; Vehicular IoT/Intelligent Transportation, these are studies addressing security for connected cars and traffic systems; Internet of Medical Things/Healthcare IoT, these are studies centered on protecting sensitive patient data and medical devices; and General/Mixed IoT Environments, this involves studies that did not focus on a specific application domain. This distribution highlights the diverse security challenges across the IoT landscape and highlights a potential research gap in less-explored domains like agriculture or supply chain IoT.

A significant finding from our overview is the diversity of datasets used in the included studies, as presented in Table 9. While a substantial number of studies utilized well-known public datasets like NSL-KDD and UNSW-NB15, a portion of the research relied on custom-generated datasets. Custom-generated datasets refer to network traffic data generated by the author rather than publicly available dataset. Studies using custom-generated datasets (*n* = 6) are grouped separately. For these entries, standard descriptors such as feature count or total records are marked ‘NA’ (Not Applicable) as they are typically not generalizable or consistently reported across studies. Furthermore, a key observation is that most studies used generic cybersecurity datasets to train what they propose as domain-specific IoT intrusion detection systems. This highlights a shortage of specialized IoT datasets for intrusion detection studies within the current body of literature.

Security challenges in IoT vary across domains like industrial, smart city, and healthcare, influenced by operational context, system scale, and environmental constraints. For instance, agricultural IoT systems face unique vulnerabilities such as unauthorized access and data tampering due to limited infrastructure and sensor constraints [96,97]. Similarly, IoT integration in supply chains increases interdependencies and the attack surface, where insecure device provisioning and third-party risks threaten data integrity and system resilience [98]. Despite the significance of these domains, our review shows that explainable deep learning-based intrusion detection research mainly targets generic or industrial IoT environments, with little focus on context-specific threat modeling and domain-tailored evaluations in agriculture or supply chain settings. These gaps highlight the need for contextualized security solutions and domain-specific evaluation frameworks to ensure robust intrusion detection across all IoT applications.

**Table 9:** IoT Application domains and the distribution of datasets used (*n* = 129 included studies). Datasets were categorized based on authors’ explicit identification in each study’s methodology section.

IoT Application Domains	Dataset Used	Year	Ref. Count	# of Features	Total Records	Source Ref.	Key Characteristics & Relevance
Industrial IoT	WUSTL-IIoT-2021	2021	3	41	1,194,464	[99]	Specifically designed for IIoT, includes PLC data and a wide variety of attacks (e.g., DDoS, Reconnaissance, Spoofing).
	X-IIoTID	2021	1	67	820,834	[100]	A recent benchmark dataset for IIoT with both network traffic and device-level logs from various IoT devices.
Vehicular IoT/Intelligent Transportation	Car-Hacking (CAN Intrusion)	2017	2	11	4,613,909	[101]	Focuses on in-vehicle networks; contains raw CAN bus traffic with injection attacks (e.g., DoS, Fuzzy, Spoofing).
	VeReMi	2018	1	13	3,194,808	[102]	A dataset for misbehavior detection in Vehicle-to-Everything (V2X) communication, simulating false information attacks.
Cybersecurity	CICIDS2017/2018	2017/2018	12	80	2,830,743	[103]	Not IoT-specific but widely used as a baseline. Contains benign and modern attack traffic, useful for comparison.
	TON-IoT	2020	11	83	22.3 M	[104]	Comprehensive data from a smart home/office network, including Windows and Linux system logs alongside IoT sensor data.
	NSL-KDD	2009	27	43	148,517	[105]	An improved version of the KDD’99 dataset that can still be used for historical comparison.
	UNSW-NB15	2015	24	49	2,540,044	[106]	A popular alternative to CICIDS, featuring a mix of modern synthetic activities and attacks
	BoT-IoT	2019	12	46	73,360,900	[107]	Blends legitimate IoT traffic with DDoS, DoS, Recon, and Theft attacks.
IoMT/Healthcare	CICIoMT	2024	2	45	8,234,515	[108]	A modern dataset with network traffic from real medical devices (insulin pumps, pacemaker simulators).
	N-BaloT	2018	6	115	849,234	[109]	Focuses on botnet attacks (Mirai, Bashlite) captured from 9 real IoT devices. Excellent for device-specific botnet detection.
Smart cities IoT	CIC IoT 2022	2022	9	45	47 M	[110]	A new dataset from the Canadian Institute for Cybersecurity, designed to address gaps in previous IoT datasets.
	CIC-BoT-IoT	2022	5	80	3,668,045	[111]	A newer dataset designed to address gaps in previous IoT datasets.
	CICIoT2024	2024	2	84	5 M	[108]	A newer dataset from the Canadian Institute for Cybersecurity, with network traffic extracted using different extraction approaches.
General/Mixed IoT Environment	IoT-23	2020	6	19	325 M	[112]	20 malware and 3 benign captures from IoT devices. Valued for its real malware traffic and variety of devices.
	Custom-generated datasets	NA	6	NA	NA	[Author-generated]	NA

Custom-generated datasets—Datasets generated by the authors, NA—Not applicable.

### 5.2. RQ1: XAI Technique Trade-Offs

This RQ consists of two sub-questions: (a) How do XAI techniques impact detection accuracy? and (b) How do XAI techniques impact computational efficiency?

#### 5.2.1. Findings

**Figure 5:**
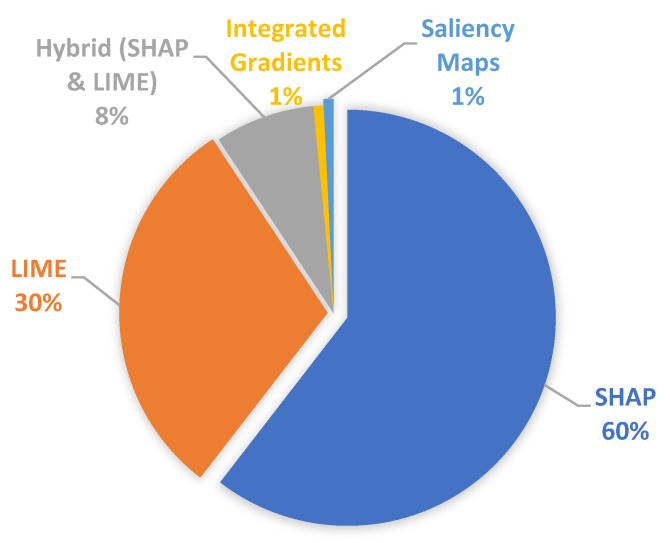
Distribution of XAI Techniques used in the included studies (*n* = 129). Techniques were coded based on the primary XAI method explicitly implemented and evaluated in each study.

Analysis of the included studies revealed that SHAP (SHapley Additive exPlanations) and LIME (Local Interpretable Model-agnostic Explanations) were the most commonly applied post hoc explainability techniques, accounting for 98% of studies, respectively, as depicted in Figure 5. Other techniques included Integrated Gradients (1%) and saliency maps (1%).

Impact on Detection Accuracy (RQ1a): Across all 129 studies, the integration of post hoc XAI techniques exhibited minimal to no negative impact on the predictive accuracy of underlying deep learning models. Specifically, across 129 studies, we found no reported instances where authors attributed a drop in core detection metrics (accuracy, precision, recall) to the integration of SHAP or LIME. If XAI integration were fundamentally damaging to accuracy, it would likely appear as a noted limitation or a trade-off discussed in the literature. Its absence is a significant finding. This is supported by a key observation from the reviewed literature. It aligns with the fundamental characteristic of post hoc methods: they analyze an already-trained model without altering its parameters or inference function. Crucially, we found no reported evidence where core detection metrics were degraded by the addition of XAI tools like SHAP or LIME.

Impact on Computational Efficiency (RQ1b): In contrast to the minimal to no negative accuracy impact, there is a computational overhead introduced by post hoc XAI explanation generation. This overhead is frequently cited as a primary barrier to real-time deployment on resource-constrained IoT devices. Among the included studies that employed SHAP and/or LIME, 21 studies included qualitative statements acknowledging computational concerns. While direct, quantified comparisons of pre-XAI and post-XAI model latency or overhead range are absent, 16.3% (*n* = 21) of the included studies identified post hoc XAI explanation generation as a limitation that could have significant overhead on resource-constrained IoT devices.

#### 5.2.2. Insights and Implications

The findings reveal a critical decoupling between detection performance and computational cost in XAI-enhanced IDSs. Post hoc techniques preserve accuracy because they operate after model prediction, analyzing the model’s decision without altering its internal weights or forward propagation. This architectural separation ensures detection capability remains intact while introducing a separate, resource-intensive explanation generation pipeline. Integrating post hoc techniques exhibits no impact because these methods are integrated after the model has made a prediction (post hoc), analyzing the model’s output and internal state to generate an explanation. Consequently, the high accuracy often associated with deep learning models, including CNNs and LSTMs, is unaffected. This indicates that achieving model explainability does not necessarily entail a compromise in detection or classification performance. However, within the broader context of model design, there exists a trade-off between accuracy and interpretability. Several scholarly works have observed that simpler, inherently interpretable models may not attain the same level of predictive accuracy as more complex, deep learning architectures [30,41,113]. These findings underscore the importance and value of applying XAI methods to more sophisticated yet opaque models, as they facilitate interpretability without substantially sacrificing the high accuracy characteristic of advanced deep learning systems.

The additional computational cost associated with generating the explanation, especially when dealing with high-volume IoT traffic, presents a substantial impediment to the real-time deployment of XAI models on resource-constrained devices. The overhead and processing time may even become more significant when both SHAP and LIME are used for explanation. The added processing time required for generating explanations through XAI techniques introduces inherent latency. While a model may rapidly classify network packets, potentially within milliseconds, the subsequent explanation generation, particularly with methods such as SHAP, can require hundreds of milliseconds. Moreover, the computational demands associated with XAI could also lead to higher memory and energy consumption. This latency could compromise the system’s ability to respond promptly to threats. These factors collectively impact the computational efficiency of XAI and limit the feasibility of deploying XAI-enhanced IDSs directly on resource-constrained IoT devices.

### 5.3. DL Model Comparison

This RQ examines two sub-questions: (a) Which architectures achieve best detection performance? and (b) Which architectures achieve best resource efficiency?

#### 5.3.1. Findings

**Figure 6:**
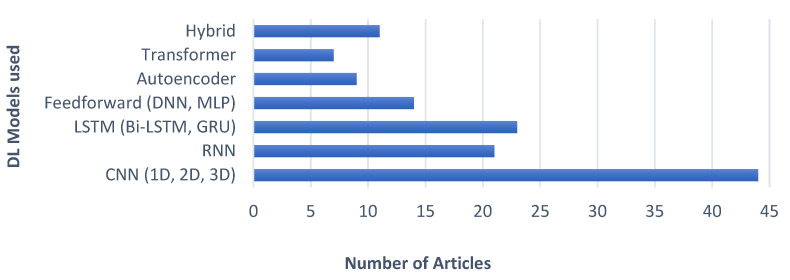
Distribution of DL models used in included studies for IoT networks (*n* = 129). Models were categorized by their primary architectural family used in each included study.

Based on the analysis of the selected studies, as shown in Figure 6, most of the included studies utilized the convolutional neural networks and their other variants, such as the one-dimensional or two-dimensional CNN, appearing in 34.1% of the studies (*n* = 44). LSTM (Bi-LSM and GRU) were employed in 17.8% (23), with RNN in 16.2% (*n* = 21). However, when LSTM (Bi-LSTM and GRU) and RNN were combined as a sequential architecture (RNN, LSTM, GRU), they were utilized in 34.1% (*n* = 44). Feedforward networks (DNN, MLP) were used in 10.8% (*n* = 14), hybrid architectures in 8.5% (*n* = 11), autoencoder in 6.9% (*n* = 9). The least used DL model is the transformer model, accounting for only 5.4% of the selected articles. Note that DL models implemented for comparison are not considered.

Detection Performance Comparison (RQ2a): For the DL architecture with the best performance metrics, we categorized the DL model utilized in the selected articles under the architectural category, with the range value for their performance metrics as shown in Table 10. We only considered these metrics, accuracy, precision, recall, and F1-score, because they are common across the selected studies. The result suggests that the autoencoder model achieves the highest detection rate with an average accuracy of 97.7% for high-dimensional IoT traffic. This is closely followed by transformer architecture with an average accuracy of 97.5%. The hybrid architectures achieve impressive performance, reflected in their high F1-score range. Furthermore, the lightweight CNN architecture exhibits the broadest range of performance and lowest minimum value across all metrics (e.g., accuracy as low as 77.5%, F1-score as low as 76%).

Resource Efficiency Comparison (RQ2b): To measure the resource efficiency of the models in the selected studies, we categorized the models used and their resource efficiency metrics, such as inference latency, throughput, energy consumption, and memory, as depicted in Table 11. However, there is a significant lack of reported efficiency metrics in the literature, as most included studies focused exclusively on algorithmic performance and validation. Consequently, our analysis could only draw conclusions from a limited subset of 12 studies (9.3% of the total) that reported at least one relevant efficiency metric. The analysis of the resource efficiency data, Table 11, reveals notable differences in performance between CPU-based and GPU-based processing. For instance, models deployed on GPUs in studies [25,114] achieved a remarkably low latency, making them strong candidates for real-time detection. In contrast, models running on standard CPUs in studies [115,116] exhibited high latency values ranging from 15 to 16.05 s. The reported latency for a Lightweight CNN was 5.9 s on a CPU in study [117]. The findings also reveal that only one study reported specific energy consumption (70 Joules for Autoencoders on GPU), and only two provided a memory footprint (146.88 MB for LSTM, 9.1 MB for Autoencoder), making thorough resource comparisons impossible.

#### 5.3.2. Insights and Implications

CNNs, particularly the 1D or 2Ds, are lightweight and provide advantages such as reduced computational complexity, faster inference, lower memory consumption, and energy efficiency when deployed in IDSs for IoT networks [50,125]. The limited adoption of the transformer model suggests its relatively limited use in DL-based IDSs for IoT networks compared to other architectures. The high performance of autoencoders is due to their strength in unsupervised learning and anomaly detection, as they are highly effective at identifying deviations from normal network behavior, thus being able to detect unknown attacks without prior knowledge of their signatures [34,126]. Hybrid architectures leverage the strengths of different architectures, such as a CNN’s capability to extract spatial features and an LSTM’s ability to capture temporal dependencies, enabling high accuracy on complex datasets. However, this superior performance typically incurs a cost, as hybrid models tend to be more computationally intensive and complex to deploy, posing a significant trade-off for resource-constrained IoT devices [127].

Moreover, the lightweight CNN architecture is one of the most suitable for deployment on resource-constrained IoT devices due to its lower computational complexity [126]. While highly optimized CNN models can achieve superior performance, their consistency is significantly lower and potentially more sensitive to factors such as hyperparameter tuning, dataset characteristics, and attack types compared to more robust but computationally intensive sequential and transformer models. These findings highlight that the architecturally best DL model for detection performance may not be the most practical or efficient for real-world IoT deployment scenarios.

Most studies neglected the critical evaluation of computational cost, energy consumption, and real-time inference capabilities necessary for IoT deployment. The observed latency differences between the processing units indicate that although various DL architectures are utilized, the choice of underlying hardware (CPU or GPU) is the most immediate bottleneck in the practical deployment of XAI-enhanced IDSs at the constrained IoT edge. Moreover, while Lightweight CNNs are theoretically suited for edge devices, the reported latency indicates that without careful optimization, even simple models can struggle to meet the low-latency demands of IoT networks.

While the available data is insufficient to definitively rank architectures for IoT deployment, it highlights that performance is highly dependent on the hardware-software configuration and that the field urgently needs standardized benchmarking that includes energy and memory profiling alongside latency.

### 5.4. XAI Evaluation Framework

This RQ examines how XAI techniques are evaluated for effectiveness and reliability. To provide answers to how XAI techniques are evaluated after deployment, we first develop an evaluation category framework, adapted from [94], presented in Table 12. This framework functions as a taxonomy tool to categorize and evaluate the methodological rigor of XAI research papers, serving both analytical and prescriptive purposes. It offers researchers and authors a hierarchical checklist to design and report evaluations, encouraging progression from basic evaluation categories to incorporating quantitative metrics, human validation, or real-world task integration to enhance validity. While existing XAI evaluation often focuses on technical metrics like faithfulness, robustness, and stability (Category C), our XAI Evaluation Framework expands this view by integrating human-centric (Category D) and application-centric (Category E) evaluation as essential, higher-level stages.

#### 5.4.1. Findings

To systematically analyze XAI evaluation practices, we applied the five-category XAI Evaluation Framework (Table 12) to all included studies. The analysis of the evaluation of XAI techniques after deployment, as depicted in Table 13, shows that 94.6% of the included studies include some form of subjective or objective evaluation of the XAI technique deployed in their study. 7 studies (5.4%) provided no metric, user study, claim, or criteria to confirm explanation correctness or utility. 117 studies (90.6%) relied on author visual inspection and subjective claims about feature importance. 5 studies (3.9%) employed rigorous metrics such as faithfulness, stability, or actionability to measure explanation quality. 0 studies (0%) involved security analysts or domain experts in controlled evaluation. 0 studies (0%) tested explanations in real-world tasks or operational scenarios. Among the 117 studies employing Category B evaluation, the most common justification patterns were: “Feature X exhibited the greatest impact while feature Y demonstrated the least influence”, “Feature X emerged as the most significant feature with a greater likelihood of classifying an instance of an XYZ attack” in studies [25,118,119].

#### 5.4.2. Insights and Implications

The near-total reliance (90.6%) on author-asserted plausibility checks (Category B) without independent validation introduces high risk of confirmation bias. Authors’ natural interpretation of the XAI outputs without objective assessment provides no empirical evidence that explanations are faithful to the model’s actual decision process or useful for security operators. The pattern “Feature X was most important” is a claim which may be due to correlated features or explanation method limitations rather than genuine causal influence on the prediction. This implies that currently, XAI evaluation in the literature is a simple, post hoc justification that lacks objective measurement. This is shown by the overwhelming prevalence of Category B, the minimal adoption of quantitative fidelity metrics (3.9% in Category C), and the complete absence of human-based assessment or application-centered testing (Categories D and E). This indicates that while the field recognizes the need to justify its explanations, it has not yet adopted scientifically rigorous methods to do so. The near-total reliance on subjective, author-claimed plausibility checks introduces confirmation bias and provides no empirical evidence that the explanations are faithful to the model, robust against manipulation, or actually useful for a security operator’s decision-making. Consequently, the practical reliability and utility of these XAI techniques in real-world IoT security operations remain largely unproven. The evaluation category framework explicitly calls attention to the higher-tier evaluation categories (C, D, E) that the field must adopt to establish explanation reliability.

### 5.5. Bottlenecks and Mitigations

This RQ synthesizes evidence from the preceding analyses to identify systemic deployment barriers and mitigation approaches. Based on the findings from RQ1–RQ3, there are some performance-related and non-performance-related issues in XAI-based IDSs for IoT networks. In what follows, we discuss the bottlenecks and provide some mitigation strategies to these issues.

#### 5.5.1. Findings: Identified Bottlenecks

1. Computational Overhead of Post hoc XAI Techniques

The main challenge in deploying XAI-enhanced IDSs in IoT networks is the significant computational overhead associated with post hoc explanation methods like SHAP and LIME, as identified in 21 studies. While deep learning models can classify data quickly, generating explanations can require vastly more processing time and resources. This is particularly true for model-agnostic techniques that rely on iterative sampling or comparisons across many data points to produce robust explanations. Consequently, this computational demand directly increases inference latency for the entire IDS pipeline, making the system unsuitable for the real-time detection and response needs of time-sensitive IoT applications, such as industrial control or vehicular networks. Furthermore, this high computational load leads to substantial memory footprint and energy consumption, which are critical limitations for resource-constrained IoT edge devices. The processes involved in generating explanations and storing feature importance data place a heavy burden on battery-powered or low-power devices, thereby accelerating battery drain and reducing system operational longevity, making true on-device XAI infeasible.

2. Lack of comprehensive computational efficiency reporting

Another significant bottleneck hindering the practical advancement of XAI-IDSs for IoT is the general lack of comprehensive reporting on computational and resource efficiency. As evidenced in this review, 12 studies reported essential metrics such as inference latency, energy consumption, or memory footprint. As a result, there is a substantial gap in understanding the real-world deployability of the proposed models. This omission means the literature is filled with solutions that may achieve high detection accuracy in controlled experimental settings but could be impractical for actual resource-constrained IoT hardware due to excessive computational demands. The impact of this reporting gap is a misdirection of research efforts and a failure to address the fundamental constraints of the IoT domain. Without standardizing the reporting of efficiency metrics, meaningful comparisons between different XAI architectures are impossible, making it difficult to identify models that truly achieve an optimal balance between performance and resources.

3. Inadequate IoT-domain-specific datasets for intrusion detection studies

The reliability of XAI-IDSs is compromised due to a prevalent lack of high-fidelity, IoT-domain-specific datasets. Most existing studies rely on either legacy, generic cybersecurity datasets (e.g., 27 studies for NSL-KDD, 24 studies for UNSW-NB15) or small, custom-generated data (6 studies). Legacy datasets were designed for traditional IP networks and fundamentally lack the unique protocol characteristics of real-world IoT systems, such as lightweight MQTT, CoAP traffic, and device-specific communication signatures. This results in models that are trained on irrelevant or incomplete feature sets, leading to misleading performance metrics that do not generalize well against actual IoT attack vectors. In addition, the absence of realistic, diverse, and large-scale IoT attack scenarios prevents models from adapting to real-world heterogeneous environments. The explanations generated by these models are tailored to the artificial conditions of the training set and become unstable when deployed in different contexts. This undermines trust in the entire XAI system, as the explanations cannot be reliably used for critical security decisions.

#### 5.5.2. Insights: Mitigations Strategies

1. To manage high computational demands, researchers should use model-specific explainability methods. Techniques like Grad-CAM for CNNs leverage internal gradients and activation maps to produce efficient explanations without requiring input perturbation or numerous model queries [30]. Additionally, XAI computation should be offloaded from constrained IoT devices to more capable platforms, such as local edge gateways, fog nodes, or local server clusters, ensuring that real-time intrusion detection remains unaffected by the explanation process.
2. To reduce resource costs of post hoc explanations, the focus should shift to architectures that are intrinsically interpretable, like attention-based models where attention weights serve as explanations [128] or that use knowledge distillation. In this approach, a complex, high-accuracy “teacher” DL model trains a smaller, faster, and more memory-efficient “student” model. The student model, which is easier to interpret, can then be deployed at the resource-constrained edge, effectively reducing inference time and overhead for generating reliable explanations [129].
3. To tackle the issue of inadequate computational reporting, the research community should first establish and adopt a minimum reporting standard for efficiency metrics. This standard should require the inclusion of inference latency, energy consumption, and memory usage. By doing so, it will create a baseline for comparability and help validate the model’s practical feasibility for constrained IoT edge environments.
4. To address the inadequacy of IoT-domain-specific datasets, a collaborative effort is needed to create and maintain large-scale, publicly available benchmark datasets derived from real-world IoT environments. This involves building heterogeneous testbeds with a variety of devices, including sensors, cameras, and smart appliances, while also recording comprehensive network traffic that encompasses a wide range of modern, IoT-specific attacks (e.g., MQTT exploits, CoAP DDoS). It is essential that these datasets accurately reflect a variety of normal behaviors to minimize false positives.

### 5.6. Unified Explainable IDS Evaluation Framework (UXIEF)

**Figure 7:**
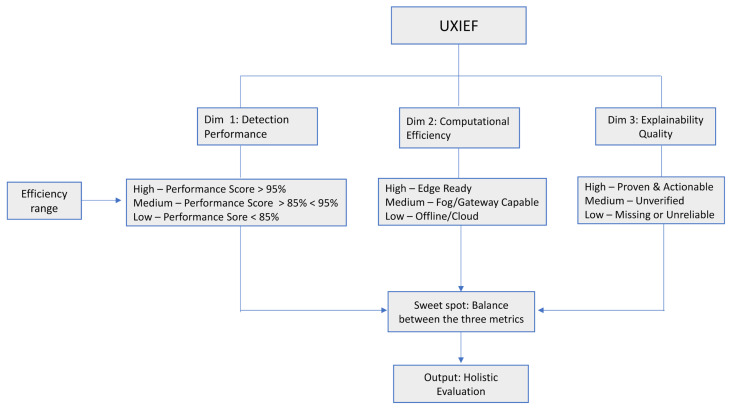
Conceptual framework for the Unified Explainable IDS Evaluation Framework (UXIEF) Trilemma, modeling the tensions between detection performance, computational efficiency, and explainability quality.

The findings from our systematic review reveal a consistent trilemma. The pursuit of high detection performance, transparent explanations, and resource efficiency exists in a state of fundamental tension. To conceptualize this, we propose the IoT-XAI Trade-off Trilemma, otherwise known as UXIEF, a three-way constraint framework that details the essential metrics and their efficiency range, as well as reflects the real-world deployment challenges in IoT shown in Figure 7.

This framework serves as a holistic design guide and strategic decision-making tool to visualize and manage the essential trade-offs involved in deploying an XAI-IDS for IoT. It emphasizes the interconnectedness of detection performance, computational efficiency, and explainability quality, encouraging a balanced approach from the beginning rather than optimizing for one aspect first. The framework helps identify the optimal sweet spot tailored to specific IoT scenarios. For benchmarking and comparison, it provides a multi-dimensional scoring system (High/Medium/Low) to evaluate different XAI-IDS studies beyond traditional metrics like F1-score. Systems can be mapped within the trade-off space to reveal their strengths and weaknesses, enabling a more nuanced assessment. It models what should be prioritized to achieve deployable systems by making the trade-offs explicit and quantifiable. To precisely contextualize UXIEF, we define the prevailing evaluation paradigm observed in our review as encompassing three dominant yet siloed practices: (i) isolated accuracy benchmarking, confined to detection metrics; (ii) siloed efficiency checks, involving reporting of computational metrics; and (iii) post hoc XAI evaluation, where explanation tools are applied and validated mostly via author-driven, qualitative plausibility checks (Category B of our XAI Evaluation Framework). These techniques function primarily as isolated diagnostic tools. UXIEF is designed to transcend this fragmented approach by mandating a simultaneous, tripartite evaluation where progress in one dimension cannot mask critical deficiencies in another, thereby directly modeling the interdependencies of the IoT IDS trilemma.

A breakdown of the three metrics and their range of efficiency, as well as their definition and implications, is described in Table 14. The detection performance measures the effectiveness of the IDS, the computational efficiency addresses the resource constraints as it relates to the underlying deep learning model architecture, while the explainability measures how good the explanation provided is.

To enable a quantitative analysis of the research landscape, we introduce a grading scheme that assigns a score (3: High, 2: Medium, 1: Low) to each included study across the three UXIEF dimensions. For studies that did not report their computational efficiency, as discussed in Section 5.3, we classified them as low efficiency. Then we applied the proposed UXIEF to grade the 129 included studies across the three dimensions with the aim to quantify and visualize the field’s current imbalances and identify strategic directions for future research.

#### UXIEF Application

To illustrate the application of the UXIEF conceptual framework (Figure 7) and the UXIEF grading criteria (Table 14), we present detailed evaluation of three representative studies to show how individual studies are mapped and scored across the three UXIEF dimensions.

**Example** **1.** *Efficient Latency on GPU (Jain et al., 2025* *[114]). This study proposes an XAI-enhanced hybrid deep learning framework for IoT device identification and attack detection.*

Detection Performance (Grade: High-3): The study reports high accuracy and F1-scores (exceeding 95%) on a modern IoT traffic dataset, meeting the criterion for High detection performance.

Computational Efficiency (Grade: Medium-2): The study reports a low inference latency of 0.0389 s (38.9 ms) for its CNN model on a GPU. While this latency is below the 100 ms threshold, the metric is reported for a GPU accelerator rather than a representative IoT edge processor. Furthermore, no memory footprint or energy consumption data is provided. This constitutes partial characterization, warranting a medium efficiency grade.

Explainability Quality (Grade: Medium-2): The study employs SHAP to provide feature importance explanations for device identification and attack classification. The explanations are presented visually and discussed qualitatively, without quantitative fidelity metrics or human-centered validation. This aligns with medium explainability quality.

UXIEF Score for [114]: (Detection: 3, Efficiency: 2, Explainability: 2) = Total: 7/9.

**Example** **2.** *Low Latency on GPU, with Memory Reporting (Ogunseyi & Thiyagarajan, 2025* *[25]). This study proposes an LSTM-based IDS optimized by the Firefly Algorithm and uses SHAP for explanations.*

Detection Performance (Grade: High-3): The study reports an accuracy of 99.65% and an F1-score of 99.7% on the IoT dataset, meeting the high-performance criterion.

Computational Efficiency (Grade: Medium-2): The study reports a very low inference latency of 0.0085 s (8.5 ms) and a memory footprint of 146.88 MB. While the latency is excellent, the memory footprint far exceeds the <10 MB edge-compatible benchmark, and the metrics are reported for a GPU, not a representative edge processor. This partial characterization, showing high performance on non-edge hardware, warrants a medium efficiency grade.

Explainability Quality (Grade: Medium-2): The study employs SHAP to generate feature importance plots, with conclusions drawn from visual inspection. No quantitative fidelity metrics or human validation are provided, fitting the medium category.

UXIEF Score for [25]: (Detection: 3, Efficiency: 2, Explainability: 2) = Total: 7/9.

**Example** **3.** *High explainability with quantitative metrics (Kalakoti et al., 2024* *[130]). This study improves the transparency of high-performance models for IoT botnet detection.*

Detection Performance (Grade: High-3): The study reports an F1-score of 99.9% on the IoT dataset, meeting the high-performance criterion.

Computational Efficiency (Grade: Low-1): The study fails to report its inference latency, memory footprint, or any computational efficiency metrics. This classifies the study into low efficiency grade.

Explainability Quality (Grade: Medium-3): The study employs both SHAP and LIME to generate feature importance plots. It further presents quantitative metrics such as faithfulness, sensitivity, and monotonicity, fitting the high category.

UXIEF Score for [130]: (Detection: 3, Efficiency: 1, Explainability: 3) = Total: 7/9.

**Figure 8:**
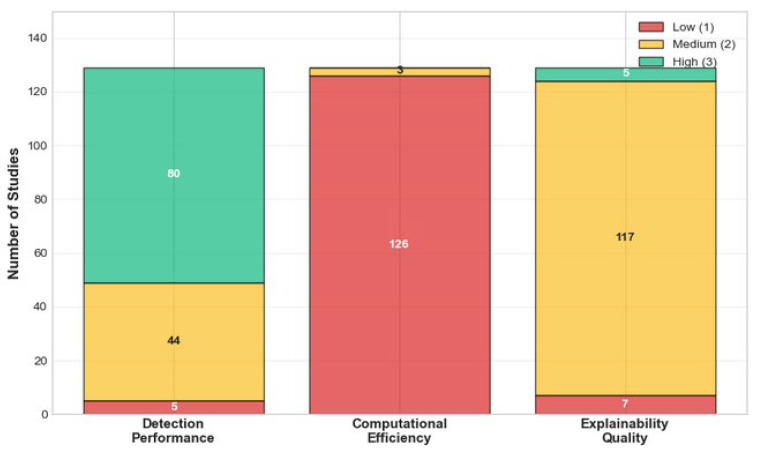
Performance distribution across UXIEF three Dimensions for the included studies (*n* = 129). Each study was graded (High = 3, Medium = 2, Low = 1) on each dimension using the criteria defined in Table 14.

These examples demonstrate the UXIEF trilemma in practice. The studies achieve high detection performance, medium to low grades for efficiency, and medium to high grades for explainability. This scoring logic, applied to all 129 studies, yields the aggregate distributions shown in Figure 8.

Our analysis, from Figure 8, reveals a huge concentration of research, as over 60% of proposed systems fall into the high-performance category characterized by high detection but low efficiency and/or medium explainability quality. This concentration in the high-performance category underscores a predominant “detection-performance-focused” mindset within the research community. While achieving high detection rates is crucial, this focus has largely come at the expense of the other two pillars of the UXIEF trilemma, creating a significant gap between proposed SOTA and the practical realities of resource-constrained IoT environments. The computational efficiency dimension reveals the most critical barrier to real-world deployment. An overwhelming majority of studies fall into the low-efficiency category, indicating that the resource footprint of the underlying deep learning model architecture often renders them impractical for IoT edge devices. This suggests that current deep learning-based IDS designs, even before considering post hoc explanation methods like SHAP and LIME, are too computationally intensive for devices with strict latency and energy constraints. The absence of studies in the high-efficiency category highlights a pressing need for novel, lightweight DL architectures and rigorous benchmarking that can provide effective intrusion detection without compromising the system’s ability to operate in real-time.

The explainability quality dimension demonstrates a field still in its infancy regarding rigorous evaluation. The dominance of the “medium” category indicates that while most studies attempt to provide explanations, they rely almost exclusively on theoretical XAI methods validated only by basic plausibility checks, with only a minimal number advancing to quantitative validation. The near-absence of studies in the “high” category, which requires the integration of objective metrics with human-centric and/or application-based evaluation, reveals a critical methodological gap. This predominant reliance on unverified explanations means the trustworthiness and actual utility of these systems for security analysts remain largely unproven, ultimately undermining the core purpose of incorporating XAI.

**Figure 9:**
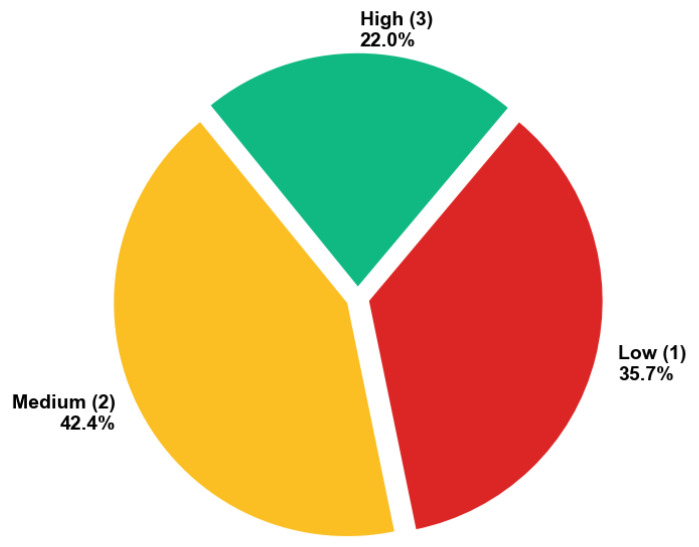
A synthesized performance distribution across UXIEF three dimensions (*n* = 129 studies). The overall score for each study was calculated as the sum of its grades (3–9) across Detection, Efficiency, and Explainability, based on the grading applied for Figure 8.

Figure 9 synthesizes the data from all three dimensions into a single visualization of the field’s overall progress. The chart confirms that “medium” performance is the most common outcome, representing solutions that make compromises across the board. More importantly, the “high” segment remains the smallest, empirically validating the UXIEF trilemma by showing that it is exceptionally rare for a study to excel in all three dimensions simultaneously. The current trajectory of XAI-based IDS research does not align with the resource-constrained IoT ecosystems. The path forward requires a fundamental shift in research priorities towards co-designing detection, efficiency, and explainability as equally critical and interconnected objectives. Future work must consciously strive to populate the vacant “sweet spot” in the UXIEF trilemma by developing systems that are not only accurate but also efficient enough for the edge and transparent enough to be truly trusted by security operators.

UXIEF’s quantifiable framing is instantiated through a structured, two-tiered analytical process. The first tier, dimensional quantification, translates textual descriptions into ordinal scores (High/Medium/Low), enabling basic aggregation and comparison (e.g., the score profile (3,1,2) quantifies an “accuracy-at-all-costs” model). The second tier is system and field-level quantification. The collective scores create a standardized dataset that enables higher-order analytics: calculating the percentage of studies achieving a “High” grade per dimension reveals field-wide imbalances and plotting studies as vectors in a trade-off space identifies strategic biases. Thus, UXIEF attempts to function as an analytical tool that converts heterogeneous study attributes into structured data, enabling systematic gap analysis and the setting of clear, multi-objective targets.

## 6. Future Research Directions

As XAI-based IDSs for IoT networks evolve, further research is required on some key areas aimed at enhancing the performance, scalability, and effectiveness of these systems in real-world deployment.

1. Holistic Co-Design of detection, efficiency, and explainability

A primary future direction is the holistic co-design of detection, efficiency, and explainability as interdependent objectives from the earliest stages of system development. This need is directly motivated by the fundamental trilemma revealed by the UXIEF, where the current research landscape shows a pronounced concentration on high detection performance at the expense of efficiency and rigorous explainability (Figure 8 and Figure 9). Current approaches treat these dimensions sequentially, leading to the severe trade-offs mapped by UXIEF. Future work must pioneer co-design methodologies where architectural choices are evaluated simultaneously against a unified optimization function that penalizes poor detection, high latency, and unreliable explanation quality equally. This requires novel design frameworks and multi-objective optimization techniques to define viable performance frontiers. Ultimately, this shift from post hoc integration to innate co-design is essential to populate the vacant “sweet spot” in the UXIEF trilemma, producing systems whose high accuracy is intrinsically coupled with the efficiency needed for the edge and the transparency required for trust.

2. Development of Resource-Efficient and Intrinsically Interpretable Architectures

Future research must prioritize the development of resource-efficient XAI techniques tailored for the IoT edge, a direct response to the computational overhead and near-total lack of efficiency reporting identified as the main deployment bottleneck (Section 5.2 and Section 5.3). This demands a fundamental shift away from computationally intensive post hoc methods like SHAP and LIME, which are unsuitable for constrained devices. The emphasis should be on creating intrinsically interpretable models, such as those with built-in attention mechanisms where explanations arise naturally from inference with minimal overhead. Furthermore, efforts should focus on optimizing model-specific explanation algorithms (e.g., Grad-CAM variants) that utilize a model’s internal state without numerous extra queries. The ultimate aim is to co-design the intrusion detection model and its explanation generator as a single, lightweight system that provides high accuracy and transparent reasoning while maintaining the low latency and power efficiency evidently absent from most current literature (Table 11).

3. Robust and Realistic IoT-domain-specific benchmark dataset

Another key priority is the creation of robust, realistic benchmark datasets specific to IoT domains, urgently needed to address the reliance on generic or custom-generated datasets that inhibit generalizable IDS development (Table 8). Current datasets often fail to capture the unique protocols, device heterogeneity, and attack vectors of real-world IoT ecosystems. Future initiatives should establish large-scale, publicly available datasets that encompass a variety of IoT protocols (e.g., MQTT, CoAP, Zigbee), a broad spectrum of modern IoT-specific attacks, and essential operational metadata. Such standardized benchmarks are vital for facilitating fair comparisons, enabling rigorous evaluation of efficiency and explainability in authentic contexts, and advancing the field toward solutions validated against real-world IoT deployment complexities.

4. Integration of Rigorous and Standard XAI Evaluation

Future research must adopt rigorous, standardized evaluation of XAI techniques that extends beyond the subjective plausibility checks dominating current practice (Table 13, Category B). This requires a framework incorporating verifiable technical metrics (Category C), such as fidelity and stability scores, to ensure explanations accurately reflect the model’s logic [131,132]. Furthermore, it must include human-centered evaluations (Category D), where security professionals perform diagnostic tasks with explanations to assess impact on decision-making speed and accuracy. Finally, application-based testing (Category E) should measure how explanations drive effective mitigation actions in simulated or real IoT environments. This approach would provide the empirical evidence currently lacking to prove an explanation’s real-world utility for security operators, moving XAI from generating explanations to objectively demonstrating their value.

## 7. Conclusions

In this study, we conduct a systematic and extensive analysis of explainable AI for intrusion detection in IoT networks. Specifically, we investigate the performance of XAI-based IDSs in IoT networks by examining the trade-offs between detection performance, the choice of DL model, resource efficiency, and the methodological rigor of evaluating explainability. Our findings reveal that there is a growing interest in the field of explainable deep learning for intrusion detection systems across diverse IoT application domains, emphasizing the importance of transparent and trustworthy security solutions for critical IoT systems.

The study provides clear answers to the guiding research questions. Regarding XAI technique trade-offs (RQ1), we conclude that while post hoc methods like SHAP and LIME do not affect the underlying model’s detection accuracy, they impose a severe and often excessive computational overhead, introducing significant latency and energy costs that hinder real-time deployment on IoT devices. Our comparison of DL architectures (RQ2) reveals that while complex models like autoencoders and hybrids achieve superior detection rates, lightweight CNNs offer a more pragmatic balance for resource-constrained environments. However, a critical lack of reported efficiency metrics in the literature prevents definitive ranking for IoT deployment. The analysis of XAI evaluation practices (RQ3) uncovered a major methodological shortcoming, which is an overwhelming reliance on subjective, qualitative plausibility checks, with a minimal presence of rigorous quantitative checks and a complete absence of human-centric, or application-based validation, leaving the practical reliability of explanations unproven. Finally, we identified key bottlenecks (RQ4), including the computational cost of post hoc XAI, the lack of efficiency reporting, and the scarcity of realistic IoT-domain datasets, and proposed mitigations such as model-specific explanations, efficiency benchmarking standards, and collaborative dataset creation.

The insights from this analysis directly informed the development of two conceptual frameworks with distinct practical applications. The XAI Evaluation Framework provides researchers with a categorical checklist to elevate the rigor of their explanation validation, moving from subjective assertion to empirical evidence. The Unified Explainable IDS Evaluation Framework (UXIEF), stemming from the observed trilemma between detection, efficiency, and explainability, serves as an actionable design tool. It enables researchers to visualize trade-offs, benchmark solutions holistically, and strategically navigate development toward balanced systems viable for the IoT edge. The application of UXIEF to the literature quantitatively confirmed that excelling in all three dimensions simultaneously is exceptionally rare, highlighting the precise challenge for future work.

Building on these, we outline some future research directions. First, a fundamental shift is required toward the holistic co-design of detection, efficiency, and explainability as interconnected objectives from a system’s inception, aiming to populate the high-performance sweet spot in the UXIEF trilemma. Second, there is a need to develop novel, resource-efficient XAI techniques, such as intrinsically interpretable architectures or highly optimized model-specific explainers, tailored for the edge. Third, the creation of robust, realistic, and public benchmark datasets capturing diverse IoT protocols and attack vectors is essential for generalizable progress. Finally, the field must adopt rigorous, standardized XAI evaluation protocols that integrate technical fidelity metrics with human-centered and application-based assessments to prove real-world utility for security operators.

This systematic study offers a coherent and comprehensive analysis of the performance of XAI-based intrusion detection systems for IoT networks, providing valuable guidance by synthesizing existing knowledge, exposing critical gaps, and proposing practical frameworks for evaluation and design. The ultimate goal is to foster the development of transparent, reliable, and deployable intrusion detection systems capable of securing the expanding IoT landscape.

## Figures and Tables

**Table 1:** Summary of the characteristics and contributions of existing related studies.

Ref	Domain	Threat/Detection	Contributions	Disadvantages
[33]	IoT	NID	Assesses traditional and IoT-specific NID High similarities and differences Evaluates supervised ML classifiers Identifies emerging trends and suggest future research directions	Lack of focus on performance and resource efficiency in resource-constrained IoT network
[20]	IoT	IDS	Assesses the strengths and drawbacks of current IDSs Emphasizes the complexity and diversity of attacks in IoT Suggests future research directions	Lack of focus on performance and resource efficiency in resource-constrained IoT network
[34]	IoT	IDS	Addresses security challenges within IoT ecosystems Examines how DL can enhance IDSs Focuses on the advantages of DL over traditional methods Emphasizes the significance of data selection for effective model training Discusses preprocessing and feature extraction techniques	Lack of focus on performance and resource efficiency in resource-constrained IoT network
[35]	IoT	IDS	Evaluates various IDSs Identifies key challenges Analyzes DL models Addresses challenges and proposed solutions the identified challenges	Lack of focus on performance and resource efficiency in resource-constrained IoT network
[36]	IoT	IDS	Addresses six key areas that connects IoT and IDS Categorizes ML and DL techniques based on their applications in IDS	Lack of focus on performance and resource efficiency in resource-constrained IoT network
[39]	XAI	NIDS	Examines XAI techniques used in NIDS Classifies threats and assesses the applicability of XAI methods Discusses challenges, limitations and potential future research directions	No focus on performance analysis of efficiency in of XAI techniques
[40]	XAI	Malware, IPS	Investigates the intersection of XAI and cybersecurity Emphasizes that AI fail to justify their results Explores the security implications of XAI systems Discusses potential attacks on XAI techniques	Lacks any form of evaluation criteria for XAI implementation
[41]	XAI	IDS, malware and spam filtering	Investigates XAI across various applications Outlines associated challenges Proposes a framework for categorizing XAI solutions	Lack of focus on unique IoT environments
[42]	XAI	Cyber threats	Highlights XAI potential to address non-interpretability Addresses the challenges of traditional AI models	Fails to explore the operational effectiveness of XAI when deployed in IoT
[43]	XAI	IDS	Investigate the integration of XAI within IDS Highlights the need for systems with interpretable insights Discusses the benefits and limitations of XAI techniques in IDS applications	No focus on performance analysis of efficiency of XAI techniques
[44]	XAI	IDS	Explores various XAI methods applied to IDS Provides an overview of current explainability techniques, datasets, and evaluation criteria Outlines critical challenges in XAI with IDS	No focus on performance analysis of efficiency of XAI techniques
[45]	XAI	Cyber threats	Investigates the application of XAI in cybersecurity Identifies several XAI techniques used in cybersecurity	No focus on performance analysis of efficiency of XAI techniques
[46]	XAI	Malware, IDS, botnet detection	Discusses the integration of XAI techniques Explores recent developments in XAI tools Addresses ethical, regulatory, and technical challenges	No focus on performance analysis of efficiency of XAI techniques
[47]	XAI	Cyber threats	Distinguishes between local and global explanation methods Identifies recent research and future directions Identifies key research opportunities	Lack of focus on unique IoT environments

**Table 2:** Comparison of detection accuracy metrics.

Metrics	Formula	Explanation	Relevance
Accuracy	(TP + TN)/(TP + TN + FP + FN)	Measures the proportion of overall correctness	Commonly used, but can be misleading in imbalanced datasets
Precision	TP/(TP + FP)	Measures the proportion of correctly identified positive cases among all positive predictions	Minimizes false alarms, which is important for security operations
Recall (Sensitivity)	TP/(TP + FN)	Measures the proportion of correctly identified positive cases among all actual positive cases	Minimizes missed attacks, which is critical for security
F1-Score	2 × (Precision × Recall)/(Precision + Recall)	Measures the harmonic mean of precision and recall, balancing both	Used for balancing between false alarms and missed detections
Specificity	TN/(TN + FP)	Measures the proportion of correctly identified negative cases among all actual negative cases	Minimizes misclassifying normal traffic as malicious

**Table 3:** Description of computational overhead metrics.

Metrics	Explanation	Relevance
Latency/Inference Time	The time taken for the IDS to process a single network packet or flow and provide a detection	It is crucial for real-time threat detection and response in an IoT network
Throughput	This is the number of packets or flows processed per unit of time	This indicates the system’s capacity to handle a high volume of IoT traffic
Memory Usage	The amount of memory required by the DL model during operation	It is a direct constraint for low-memory IoT devices and embedded systems.
Energy Consumption	This is the power drawn by the IDS components (CPU, memory) during operation	It is crucial for battery-powered IoT devices and for sustainable large-scale deployments.

**Table 4:** Description of explainability quality metrics.

Metrics	Explanation	Relevance
Faithfulness/Fidelity	It shows how accurately the explanation reflects the actual reasoning process of the black-box model	It is essential for trust and avoiding misleading explanations
Comprehensibility/Understandability	It is concerned with how easy it is for a human (e.g., a security analyst) to grasp the explanation. Whether the explanation is presented in an intuitive way	It directly impacts the usability and actionability of the NIDS
Actionability/Utility	It shows how the explanation provides insights that enable a security professional to take effective action	It is concerned with the ultimate goal of XAI in security, i.e., to empower humans in decision-making
Stability/Consistency	It shows whether similar inputs yield similar explanations or if minor changes in input drastically alter the explanation	It confirms that unstable explanations are confusing and untrustworthy
Specificity/Granularity	It shows how detailed and precise the explanation is. Whether it points to specific features or general patterns	It confirms that more specific explanations are often more actionable

**Table 5:** Research questions (RQs) guiding the systematic review.

RQ Areas	RQs	Sub-Questions
RQ 1 (XAI Technique Trade-offs)	How do XAI techniques impact the detection accuracy and computational efficiency of DL-based IDSs in IoT networks?	How do XAI techniques impact the detection accuracy of DL-based IDSs for IoT networks?
		How do XAI techniques impact the computational efficiency of DL-based IDSs for IoT networks?
RQ 2 (Model Comparison)	Which explainable DL architectures achieve the best detection performance and resource efficiency for high-dimensional IoT traffic?	What explainable DL architectures achieve the best detection performance for high-dimensional IoT traffic?
		Which explainable DL architectures achieve the best resource efficiency for high-dimensional IoT traffic?
RQ 3 (XAI evaluation)	How are post hoc XAI techniques (e.g., SHAP, LIME) evaluated for their effectiveness and reliability in explaining DL-based IDS decisions within IoT security contexts?	
RQ 4 (Bottleneck and mitigation)	What are the bottlenecks limiting the deployment of explainable DL-based IDSs in large-scale IoT networks, and how can they be mitigated?	What are the bottlenecks limiting the deployment of XDL-based IDSs in large-scale IoT networks?
		What are some of the mitigations to these bottlenecks?

**Table 6:** Search strings and keywords used for the systematic literature search across five academic repositories.

Database	Search String with Field Restrictions	Notes
IEEE Xplore	(“Document Title”: “explainable AI” OR “Document Title”: XAI OR “Document Title”: “interpretable AI” OR “Abstract”: “explainable AI” OR “Abstract”: XAI) AND (“Document Title”: “intrusion detection” OR “Abstract”: “intrusion detection” OR “Document Title”: IDS OR “Abstract”: IDS) AND (“Document Title”: “internet of things” OR “Document Title”: IoT OR “Abstract”: “internet of things” OR “Abstract”: IoT) AND (“Abstract”: performance OR “Abstract”: evaluation OR “Abstract”: metrics)	Field-specific search in title and abstract
ACM Digital Library	[[Title: “explainable AI”] OR [Title: XAI] OR [Abstract: “explainable AI”]] AND [[Title: “intrusion detection”] OR [Abstract: “intrusion detection”]] AND [[Title: “IoT”] OR [Title: “internet of things”] OR [Abstract: IoT]] AND [[Abstract: performance] OR [Abstract: evaluation]]	Advanced search with field specifications
Scopus	TITLE-ABS-KEY ((“explainable AI” OR XAI OR “interpretable AI” OR SHAP OR LIME) AND (“network intrusion detection system” OR “intrusion detection system” OR NIDS OR IDS OR “anomaly detection”) AND (“internet of things” OR “industrial internet of things” OR IoT OR IIoT OR “IoT networks”) AND (performance OR evaluation OR metrics OR robustness OR “computational overhead” OR latency OR energy OR memory))	Advanced search with field specifications
Google Scholar	allintitle: (“explainable AI” OR XAI OR “interpretable AI”) AND (“intrusion detection” OR IDS) AND (IoT OR “internet of things”) AND (performance OR evaluation)	Limited to first 2800–3000 results; allintitle restricts to title field
Springer	(title: (“explainable AI” OR XAI OR “interpretable AI”) OR abstract: (“explainable AI” OR XAI)) AND (title: (“intrusion detection” OR IDS) OR abstract: (“intrusion detection”)) AND (title: (IoT OR “internet of things”) OR abstract: (IoT OR “internet of things”)) AND abstract: (performance OR evaluation OR metrics)	SpringerLink advanced search with field operators

**Table 7:** Inclusion and Exclusion Criteria applied for study selection in the systematic review.

Notation	Criteria
Inclusion	The primary purpose of the study must be related to detecting intrusions or anomalies. The IDS proposed or analyzed must employ deep learning techniques. This explicitly includes, but is not limited to, models such as Convolutional Neural Networks, Recurrent Neural Networks, Long Short-Term Memory networks, Gated Recurrent Units, Autoencoders, and Transformer-based models. The study must explicitly integrate explainability/interpretability mechanisms for the DL-based IDS. This includes post hoc techniques, intrinsic/ante hoc interpretable models. The IDS must be designed, evaluated, or specifically discussed in the context of IoT networks, devices, or traffic. The study must present some form of empirical results, performance metrics, or analytical discussion. The article must be a peer-reviewed journal article or a conference paper.
Exclusion	Review and survey articles are excluded. Articles not written in English. Articles that are not between 2018 to May 2025. Absence of empirical results. Articles utilized ML algorithm and not DL.

**Table 8:** Quality assessment criteria for evaluating explainable DL-based IDS studies.

Dimension	Criterion	Evaluation Questions	Scoring Guidance
Methodological Rigor	QA1: Clear Research Objectives	Are the study objectives clearly defined and aligned with explainable IDS development or evaluation?	2 = Explicit research questions or hypotheses stated; 1 = Objectives implied but not formally stated; 0 = Objectives unclear or absent
	QA2: Appropriate Methodology	Is the DL architecture and XAI technique appropriately selected and justified for the stated IoT security problem?	2 = Clear justification with comparison to alternatives; 1 = Selection stated but not justified; 0 = No rationale provided
Reporting Quality	QA3: Experimental Design	Are experimental procedures (data preprocessing, train-test split, cross-validation, hyperparameters) clearly documented?	2 = Fully reproducible design with all details; 1 = Some details provided but gaps exist; 0 = Insufficient documentation for replication
	QA4: Performance Metrics Reporting	Are detection performance metrics comprehensively reported (accuracy, precision, recall, F1-score, with actual values)?	2 = ≥4 metrics with numerical values; 1 = 2–3 metrics reported; 0 = ≤1 metric or only qualitative claims
Relevance	QA5: Alignment with Research Questions	Does the study directly address at least one of our research questions (XAI trade-offs, model comparison, XAI evaluation, or deployment challenges)?	2 = Directly addresses ≥2 RQs with empirical evidence; 1 = Addresses 1 RQ with limited evidence; 0 = Tangential or no clear alignment
Validity	QA6: XAI Implementation Rigor	Is the XAI technique implemented and validated (not just mentioned), with explanation outputs presented?	2 = Full implementation with validation and example outputs; 1 = Implementation without validation; 0 = Only mentioned conceptually

**Table 10:** DL models and their detection performance metrics as reported in the included studies. Ranges represent the minimum and maximum values for each metric reported across all studies using a given architectural category (*n* = 129).

Architecture Category	DL Models	Accuracy (%) Range	Precision (%) Range	Recall (%) Range	F1-Score (%) Range
Lightweight CNN	1D/2D/3D-CNN	77.5–99.9	78.7–99.8	73.4–99	76–98.8
Sequential Architectures	RNN, LSTM, GRU	87–99.9	83–100	84–100	88–99.9
Feedforward architectures	DNN, MLP	83.1–99.2	70–99.3	84.9–100	88.8–99.2
Dimensionality reduction	Autoencoders	95.4–100	94.8–100	97.2–99.9	96–100
Transformer/attention-based	Vanilla Transformer, ViT	95.1–99.9	95–99.9	95–99.9	95–99.9
Hybrid architecture	CNN + LSTM, CNN + BiLSTM CNN + GRU, DNN + LSTM	92.5–99.9	92–100	91.0–100	90.7–99.9

**Table 11:** DL models with resource efficiency metrics reported in a subset of included studies (*n* = 12 out of 129, 9.3%).

Ref	Architecture Category	Specific DL Models	Processing Unit	Latency (s) Avg	Throughput (s) Avg	Energy Consumption (Joules)	Memory (MB) Avg
[117]	Lightweight CNN	2D CNN	CPU	5.9	-	-	-
[114]		2D CNN	GPU	0.0389	-	-	-
[25]	Sequential Architectures	LSTM	GPU	0.0085	-	-	146.88
[115]		LSTM	CPU	218	-	-	-
[118]		GRU	GPU	16.15	-	-	-
[119]		LSTM	GPU	0.469	-	-	-
[116]	Feedforward architectures	DNN	CPU	15	-	-	-
[120]		DNN	GPU	2.9	-	-	-
[121]		MLP	CPU	12.42	-	-	-
[122]	Dimensionality reduction	Autoencoder	CPU	38	-	-	-
[123]		Autoencoder	GPU	30	-	70	9.1
[124]	Hybrid Architecture	LSTM + CNN	CPU/GPU	8.4/2.1	-	-	-

“-” indicates the metric was not reported for that study.

**Table 12:** Conceptual XAI Evaluation Framework: Categories and definitions used to classify the rigor of explanation evaluation in the included studies (*n* = 129).

Category	Evaluation Types	Meaning
A	No Explicit evaluation	The included study provides no metric, user study, claim, or criteria to confirm if the explanation is correct or useful (i.e., no evaluation).
B	Qualitative/plausibility check	The author visually inspects the explanation and makes a claim such as “features X and Y were the most important features”.
C	Quantitative/fidelity metrics	The study uses metrics such as faithfulness/accuracy, stability, actionability, etc., to measure the technical quality of the explanation.
D	Human-based	The explanation is evaluated by humans, such as a network analyst or cybersecurity professional, in a controlled environment.
E	Application-based	The explanation is tested in a real-world task, such as using the explanation to guide a mitigation action.

**Table 13:** Evaluation of XAI techniques after deployment across the included studies (*n* = 129). Counts represent the number of studies whose evaluation approach matched each category (A–E) defined in Table 12.

Evaluation Category	Option (Y/N)	No of Studies
A	Yes	7
	No	122
B	Yes	122
	No	7
C	Yes	5
	No	124
D	Yes	-
	No	129
E	Yes	-
	No	129

“-” indicates the evaluation approach was not utilized in the study.

**Table 14:** A description of the dimensions and their efficiency range for a holistic evaluation of the IoT-XAI trade-off trilemma (UXIEF). These criteria were used to assign the High/Medium/Low grades.

Dimension 1: Detection Performance		
Sub-Category	Definition & Criterion	Implications
High	Performance metric scores > 95% on public and recent IoT dataset	Demonstrates state-of-the-art security effectiveness
Medium	Performance metric scores between 85% and 95% on standard datasets	Acceptably suitable for non-critical systems
Low	Performance metric scores < 85% or evaluated on custom, non-replicable datasets	Indicates potential real-world unsuitability
Dimension 2: Computational Efficiency		
Sub-Category	Definition & Criterion	Implications
High efficiency: Edge-Ready	Model inference latency is optimized for real-time processing (e.g., <100 ms per sample) and/or minimal memory footprint (e.g., <10 MB) suitable for basic IoT sensors	Ideal for real-time, on-device deployment where real-time response and battery longevity are critical.
Medium efficiency: Fog/Gateway Capable	Model demonstrates moderate resource requirements or moderate memory footprint (within 10–100 MB).	Suitable for IoT gateways or fog nodes that aggregate traffic from multiple devices but may struggle with high-velocity streams.
Low efficiency: Offline/Cloud-dependent	Model requires significant computational resources (High GPU/CPU usage, >100 MB memory) or efficiency metrics are entirely omitted.	Restricted to offline analysis with little to no consideration for deployment. Generally impractical for resource-constrained IoT edge devices.
Dimension 3: Explainability Quality		
Sub-Category	Definition & Criterion	Implications
High: Proven & Actionable	Rigorously tested for accuracy and usefulness with both metrics and human or application evaluation.	High trustworthiness with objectively verified and demonstrably useful for human tasks.
Medium: Unverified	Uses standard XAI methods, but only checked for basic plausibility, not real-world value.	Provides a baseline for interpretability but offers no real-world utility.
Low: Missing or Unreliable	No explanations, or they are purely descriptive with no proof of being correct or helpful.	Offering no actionable guidance for security analyst.

## Data Availability

The original contributions presented in this study are included in the article. The information extracted from all the 129 included studies is available on GitHub (https://github.com/TaiwoBlessyn/Performance-Analysis-XDL-IDS-IoT, accessed on 2 January 2026).
